# Effect and application of cryopreserved three‐dimensional microcardiac spheroids in myocardial infarction therapy

**DOI:** 10.1002/ctm2.721

**Published:** 2022-01-29

**Authors:** Soon‐Jung Park, Hyeok Kim, Sunghun Lee, Jongsoo Kim, Taek‐Hee Jung, Seong Woo Choi, Bong‐Woo Park, Sun‐Woong Kang, David A. Elliott, Edouard G. Stanley, Andrew G. Elefanty, Kiwon Ban, Hun‐Jun Park, Sung‐Hwan Moon

**Affiliations:** ^1^ Department of Medicine Konkuk University School of Medicine Seoul Korea; ^2^ Research Institute T&R Biofab Co. Ltd Siheung Korea; ^3^ Department of Medical Life Science, College of Medicine The Catholic University of Korea Seoul Korea; ^4^ Division of Cardiology, Department of Internal Medicine, Seoul St. Mary's Hospital The Catholic University of Korea Seoul Korea; ^5^ Department of Biomedical Sciences City University of Hong Kong Kowloon Hong Kong SAR; ^6^ Department of Surgery, Wexner Medical Center Ohio State University Columbus Ohio USA; ^7^ Department of Physiology, Department of Biomedical Sciences, College of Medicine Seoul National University Seoul Korea; ^8^ Research Group for Biomimetic Advanced Technology Korea Institute of Toxicology Daejeon Korea; ^9^ Monash Immunology and Stem Cell Laboratories Monash University Clayton Victoria Australia; ^10^ Cell Death Disease Research Center College of Medicine The Catholic University of Korea Seoul Korea

To the Editor:

Heart failure remains a leading cause of death worldwide. It exerts a severe social burden, particularly in a global ageing population. Despite the clinical significance of heart failure, currently available therapeutic strategies, including heart transplantation and left ventricular assist device, are not realistic owing to several limitations and drawbacks.^1^, ^2^ Therefore, in the past few decades, cell‐based cardiac regeneration therapy has emerged as a promising alternative form of intervention.^3^ Among several potential cell candidates, cardiomyocytes derived from human pluripotent stem cells (hPSC‐CMs) have been suggested as the next most promising cell type to treat heart diseases, as they are the most similar to primary human CMs. hPSC‐CMs display a distinct cardiac phenotype featuring spontaneous contraction, coupling of cardiac excitation and contraction, and the expressions of cardiac‐specific genes, ion channels and structural proteins. However, major issues, including the heterogeneity of hPSC‐CMs, may cause tumourigenicity and arrhythmia may affect the long‐term storage of hPSC‐CMs, and cause poor survival, retention and engraftment of hPSC‐CMs in vivo following implantation into diseased hearts. These issues must be resolved before pursuing clinical application.^4^ Here, we aimed to develop a comprehensive protocol in order to overcome the aforementioned obstacles.

First, we identified the cluster of differentiation 71 (CD71; also known as transferrin receptor protein 1, TFR‐1) surface protein^5^ as a putative marker to generate a homogeneous population of functional hPSC‐CMs. To achieve this, we manually collected contracting green fluorescent protein (GFP)‐positive CMs derived from the NKX2.5^eGFP/w^ reporter human embryonic stem cell (hESC) line^6^ (Figure S1A,B, Supporting Live Image S1). They express GFP signal upon microscopically evident expression of NKX2.5 (NK2 homeobox 5), an established markers for cardiac progenitor cells (Figure S1C,D). The hESC cell line has been widely used as a reporter cell line to discover surface protein markers, including signal regulatory protein alpha (SIRPA).^7^ Subsequently, several contracting GFP^+^ hESC‐CMs and hiPSC‐CMs were used to perform a single‐cell array and finally identify CD71/TFR‐1 (Figure S1E–I, Supporting Live Images S2–S, 4). Studies have verified the functional roles of CD71 in the heart.^8^ Here, a series of characterisation experiments revealed that CD71^+^ hESC and hiPSC‐CMs (Figure 1A,B and Figure S1A–C, Supporting Live Images S5–S, 7) expressed significant amounts of CM‐specific markers, including alpha‐actinin and cardiac troponin T, and exhibited the ventricular type of action potential (≥65%) (Figure 1C and Figure S2C–F). Importantly, CD71^+^ hiPSC‐CMs displayed mature phenotypes when cultured for more than 70 days (Figure S2G,H, Supporting Live Images S8 and S9).

**FIGURE 1:**
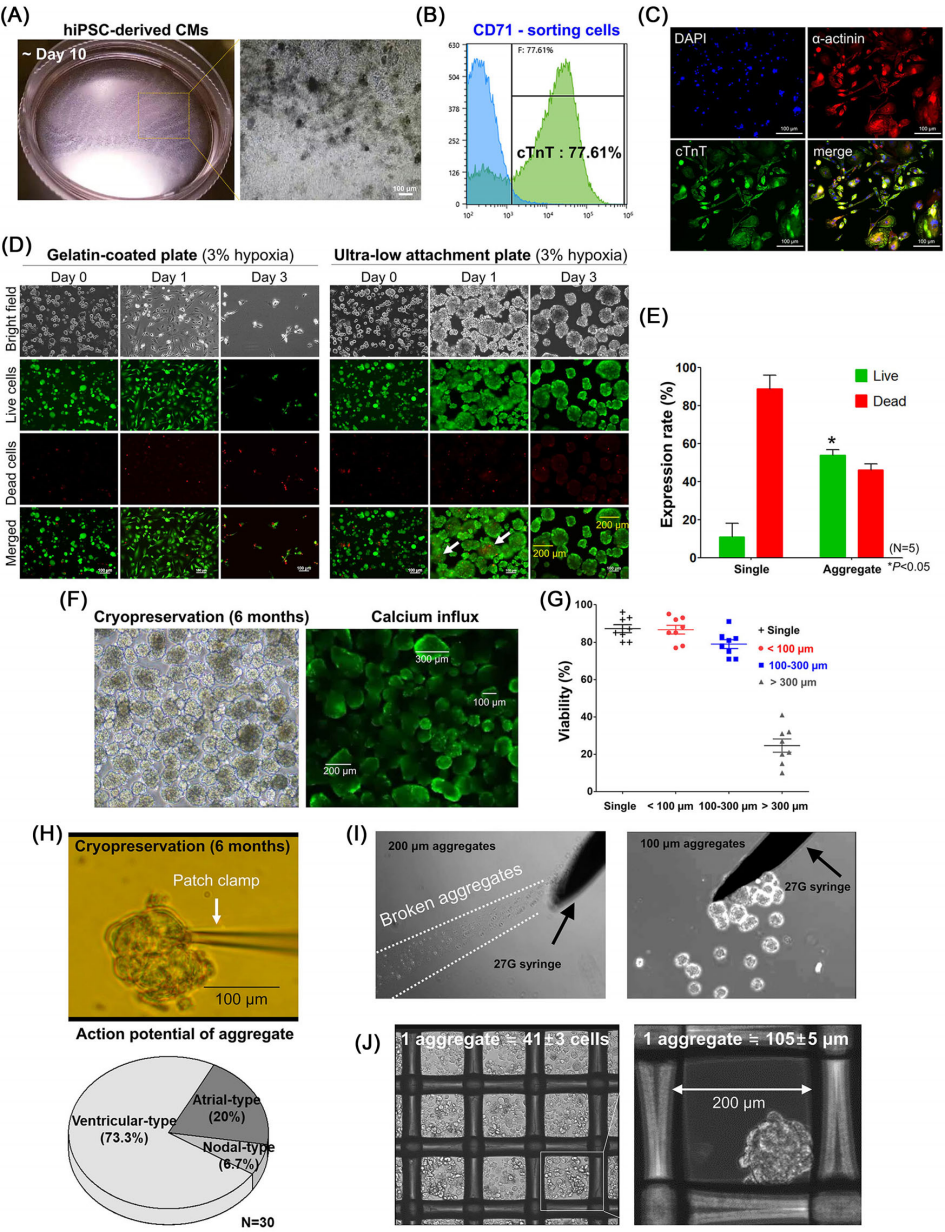
Generation of cardiomyocytes derived from human induced pluripotent stem cells. (A) Morphology of hiPSC‐CMs. (B) Flow cytometric analysis for the expression of cTnT in CD71^+^ cells. (C) Immunocytochemistry for cTnT and α‐actinin on CD71^+^ cells. Scale bars: 100 μm. (D–H) Hypoxic conditions, and cryopreservation cardiomyocyte aggregates characterisation. (D) Survival analysis of single cardiomyocytes cultured for 3 days in gelatin‐coated and ULA dish. White arrow indicates dead aggregates on day 3. Scale bars: 100 μm. (E) Cell survival rate on day 3. Data are represented as the mean ± SEM. *n* = 5 biologically independent samples per group. (F) hiPSC‐CMs cryopreservation and thawing after 6 months. Staining of different sizes of aggregates by calcium influx staining. (G) After thawing, >300 μm‐sized aggregates had the lowest survival rate (approximately 20%). (H) Electrophysiological analysis of thawed aggregates. (I) Morphology of 100‐μm aggregates was maintained while passage through a 27G syringe (left panel). The 200‐μm aggregates disintegrated while passing through a 27G syringe (right panel). (J) Formation of homogeneous aggregates using spheroid forming dish. 105 ± 5 μm aggregates were formed by plating 41 ± 3 single cells

Next, to develop an effective strategy for the long‐term preservation of hiPSC‐CMs for further clinical applications, we examined a form of hiPSC‐CM aggregate that we named ‘cryopreservable microcardiac spheroids’. Considering that the retention and engraftment of hiPSC‐CM aggregates were substantially higher in the hearts in which myocardial infarction (MI) was induced, we assumed that microcardiac spheroids could be an optimal system to induce higher survival following the prolonged storage of hiPSC‐CMs and eventual implantation in MI hearts. We observed that the microcardiac spheroids were uniform in size (100–200 μm diameter) and exhibited significantly higher survival rates in 3% hypoxic conditions lasting 7 days compared with single hiPSC‐CMs or larger hiPSC‐CM aggregates (>300 μm in diameter) (Figure 1D,E and Figure S3A–C). Furthermore, an analysis of postthaw survival revealed that 80% of the frozen microcardiac spheroids (<300 μm in diameter) remained viable after 6 months of cryopreservation in liquid nitrogen (Figure 1F–H, Supporting Live images S10–S, 13). We speculate that the substantial expression of conexin 43 protein within microcardiac spheroids (due to the proximal intracellular contact of hiPSC‐CMs) to maintain the integrity of the spheroids as a whole is the underlying mechanism for the higher survival rate in a hypoxic environment and during the physical stress of freezing.

Finally, the therapeutic efficacy of cryopreserved microcardiac spheroids (Figure 1I,J and Figure S4 Supporting Live Images S14–S, 17) was analysed in a rat model of MI^8^, ^9^ induced by ligating the left anterior descending artery (Figure S5, number of injected cells: 1 × 10^6^). Echocardiography demonstrated that cardiac function in rats treated using microcardiac spheroids was significantly higher than that in the MI control or in rats injected solely with hiPSC‐CM, as determined by ejection fraction and fractional shortening (Figure 2A–D). The results of several parameters for cardiac remodelling,^10^ such as left ventricular internal diastolic dimension and posterior wall thickness, suggested that the overall adverse cardiac remodelling in the aggregate group was significantly reduced compared with that in the other experimental groups (Figure S5A–D). Haemodynamic parameters, such as cardiac output and stroke volume, were increased in the aggregate group (Figure 2E,F and Figures S6 and S7). Similarly, Masson's trichrome staining of the cardiac tissue harvested at 8 weeks showed an area of fibrosis (%) that was substantially smaller in the aggregate group than in the other experimental groups (Figure 3A–C). Notably, transplanted microcardiac spheroids exhibited exceptional retention, engraftment and functional stability, all of which contributed to the improvement in heart function. Unexpectedly, histological analysis revealed the maturation of microcardiac spheroids in vivo in relation to the shape, structure and specific protein expression (Figure 3D–G). Maturation was not observed in single‐cell transplants, suggesting that intracellular contact and the subsequent cell–cell communication in aggregates are important for survival and drive the maturation of transplanted cells (Figure 4). Evidently, uniformity in size is also essential to induce maturation, which reduces the risk of arrhythmia.

**FIGURE 2:**
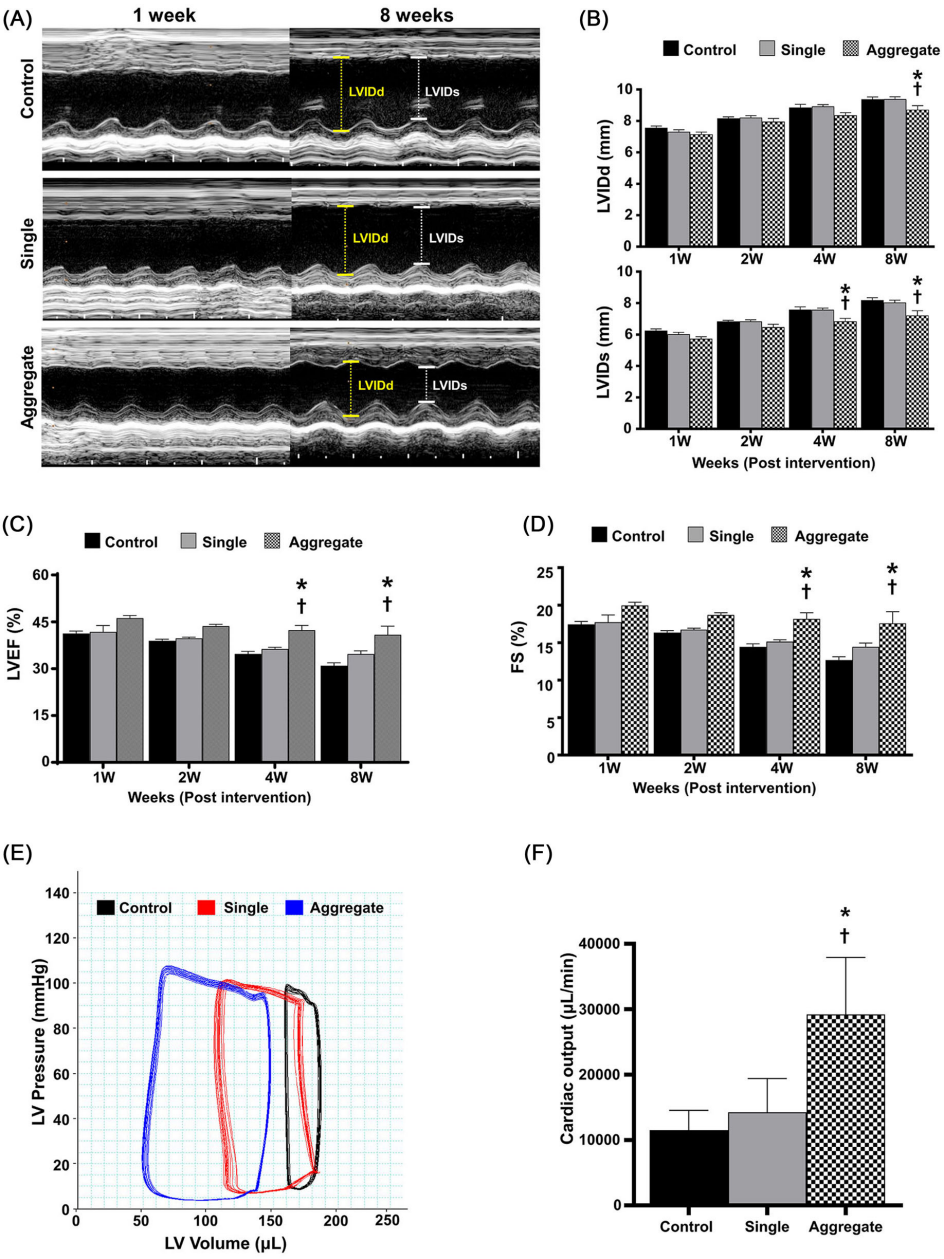
Therapeutic effects of human induced pluripotent stem cell (hiPSC)‐derived cardiomyocytes (CMs) and aggregated CMs in MI. (A) Representative images (M‐mode) of all groups at 1 and 8 weeks after transplantation. (B–D) Echocardiography was used to measure. (B) The left ventricular internal diastolic dimension (LVIDd) and left ventricular internal systolic dimension (LVISd). (C) Left ventricular ejection fraction and (D) fractional shortening. (E) Representative image of the haemodynamic pressure and volume measured using pressure volume (PV) curve at 8 weeks. (F) Haemodynamic cardiac function as measured by the cardiac output (CO). Data are expressed as the mean ± SEM. *p < .05 compared to control group, †*p* < .05 compared to the single CMs treated group; two‐way analysis of variance (ANOVA) followed by multiple comparisons with the Tukey method (*n* = 6–10 per experimental group)

**FIGURE 3:**
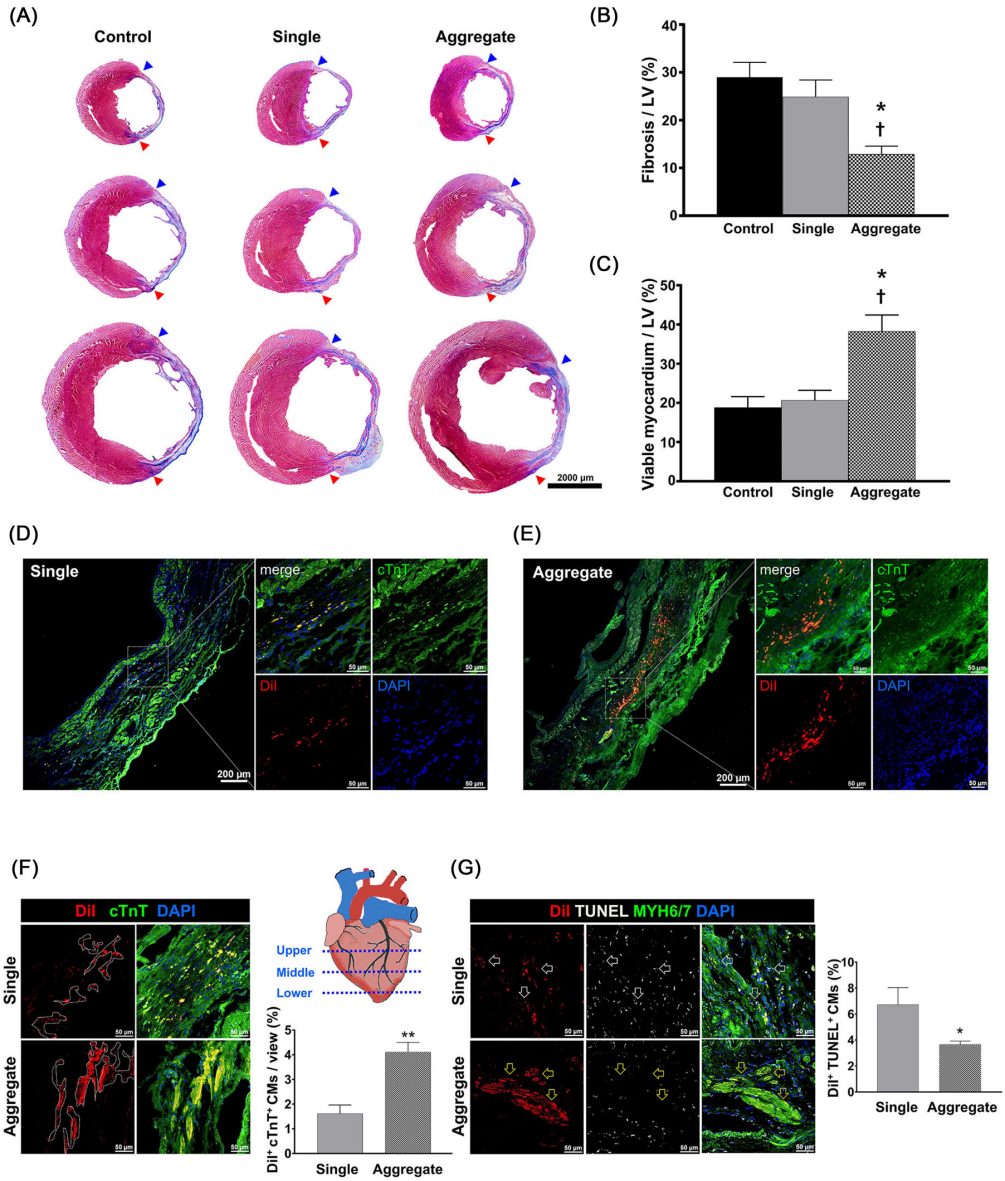
Transplantation of aggregates to MI heart improves cardiac function, cell retention and survival rate. (A) Representative images of Masson's trichrome staining from three groups show cardiac fibrosis in the heart tissues collected at 8 weeks after MI. (B) Quantitative result of fibrosis percentage in the left ventricle. (C) Quantification of the area of viable myocardium to the total area of the left ventricle. The area of fibrosis and viable tissues in the left ventricle was calculated from three different levels of each heart. One‐way ANOVA followed by Tukey's method was used for statistical analyses. Data are expressed as the mean ± SEM. *p < .05 versus control group; ^†^ *p* < .05 versus single group (*n* = 3 per group). (D and E) Representative immunostaining images of the injected DiI‐labelled CMs (red) with cTnT (green) in both single cell‐ and aggregate‐transplanted group; scale bar: 200 μm in low‐magnification panels and 50 μm in high‐magnified panels. (F) Quantification of DiI‐labelled hPSC‐CMs in the infarcted area. The area positive for both DiI (red) and cTnT (green) per five randomly selected fields from three different levels from the apex to top were quantified. **p < .01 versus single‐type group; statistical significance was evaluated using the Student's *t*‐test (*n* = 3 per group). (G) Representative images of TUNEL assay and the quantitative result of TUNEL assay. The single‐cell transplanted group showed a few TUNEL‐positive DiI‐labelled CMs (black arrows), whereas most of the DiI‐labelled CMs in the aggregate transplanted group were negative for TUNEL (yellow arrows). The TUNEL‐positive DiI^+^CMs on five randomly selected areas were counted. Scale bar: 50 μm. Student's *t*‐test was used for statistical analyses. Data are expressed as the mean ± SEM. *p < .05, **p < .01 versus single‐type group (*n* = 3 per group)

**FIGURE 4:**
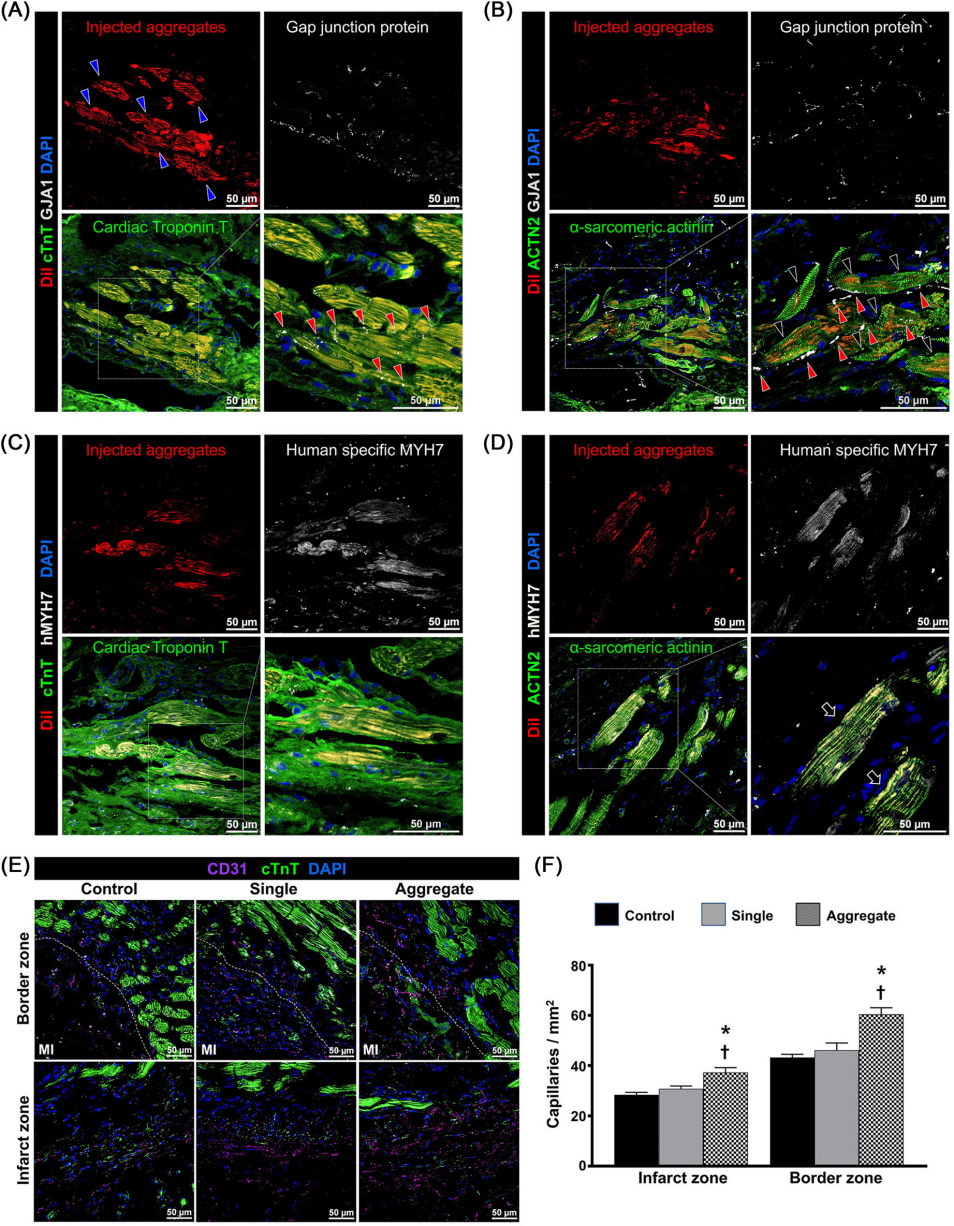
Aggregates improve engraftment and functional capacity. (A and B) Immunostaining images with DiI‐labelled CMs (red), cardiac‐specific markers (cTnT and ACTN2; green) and GJA1 (grey) showed that CA formed a gap junction with the host myocardium. Scale bar: 50 μm. (C and D) Representative immunostaining images with DiI‐labelled CMs (red), cTnT (green), ACTN2 (green) and hMYH7 (grey) verified the identity of injected hPSC‐CMs as being human origin. Scale bar: 50 μm. (E) Representative images of the capillary density. Cardiac tissue was stained with anti‐CD31 (purple) antibody to visualise the vessels in both infarct and border area at 8 weeks post cell implantation. Scale bar: 50 μm. (F) Quantitative result of capillary density. Capillaries on five randomly selected fields from three different levels were counted and the data are expressed as the number of capillaries per square millimetre. *p < .05 versus control group; ^†^ *p* < .05 versus single group (*n* = 3 per group). Statistical difference was analysed via one‐way ANOVA followed by multiple comparisons with Tukey method

In conclusion, the approach described in this study is simple and effective for the production of readily available hPSC‐CMs in clinical settings. We first enriched a pure population of contractile hPSC‐CMs based on CD71 expression. The 100‐μm microcardiac spheroids generated using CD71^+^ hiPSC‐CMs demonstrated increased survival in both hypoxia and cryopreservation, and enhanced cardiac recovery following transplantation compared to single cells. It is worth noting that the microcardiac spheroids established in this study can be preserved and stored; they exhibit superior viability under hypoxic conditions and in vivo. Thus, they are readily available for direct implantation into patients in emergencies. Despite several meaningful results, future studies examining more sophisticated implantation methods, such as the 27G NOGA catheter system^9^ and applicability to large animals, considered limitations of this study, will warrant more effective cell‐based cardiac regenerative therapy.

## CONFLICT OF INTEREST

The authors declare that there is no conflict of interest.

## Supporting information

video S1

video S2

video S3

video S4

video S5

video S6

video S7

video S8

video S9

video S10

video S11

video S12

video S13

video S14

video S15

video S16

video S17

Supporting Information
