# Supplementary material for: Effect and application of cryopreserved three‐dimensional microcardiac spheroids in myocardial infarction therapy
Source: Clin Transl Med. 2022 Jan 29;12(1):e721. doi: 10.1002/ctm2.721 (PMC8800481; doi:10.1002/ctm2.721)
Supplement: Supplementary file 18 — Supporting Information [file CTM2-12-e721-s007.docx]

**Supplementary Information**

**Effect and application of cryopreserved three-dimensional microcardiac spheroids in myocardial infarction therapy**

**Running title:** 3-D microcardiac spheroids for MI

*Soon-Jung Park****^1,2^****^#^, Hyeok Kim^3,4#^, Sunghun Lee^5#^, Jongsoo Kim****^1,6^****, Taek-Hee Jung****^1,2^****, Seong Woo Choi^7^, Bong-Woo Park^3,4^, Sun-Woong Kang^8^, David A. Elliott^9^, Edouard G. Stanley^9^, Andrew G. Elefanty^9^, Kiwon Ban^5*^, Hun-Jun Park^3,4,10*^, Sung-Hwan Moon^1,2*^*

**^1^**Department of Medicine, Konkuk University School of Medicine, Seoul, Korea

**^2^**Research Institute, T&R Biofab Co. Ltd, Siheung, Korea

^3^Department of Medical Life Science, College of Medicine, The Catholic University of Korea, Seoul, Korea

^4^Division of Cardiology, Department of Internal Medicine, Seoul St. Mary’s Hospital, The Catholic University of Korea, Seoul, Korea

^5^Department of Biomedical Sciences, City University of Hong Kong, 83 Tat Chee Avenue, Kowloon, Hong Kong SAR

**^6^**Department of Surgery, Wexner Medical Center, Ohio State University, Columbus, OH

^7^Department of Physiology, Department of Biomedical Sciences, College of Medicine, Seoul National University, Seoul, Korea

^8^Research Group for Biomimetic Advanced Technology, Korea Institute of Toxicology, Daejeon, Korea

^9^Monash Immunology and Stem Cell Laboratories, Monash University, Clayton, Victoria, Australia

^10^Cell Death Disease Research Center, College of Medicine, The Catholic University of Korea, Seoul, Korea

*#*These authors contributed equally to this work.

^*^Corresponding authors:

Correspondence to Kiwon Ban, PhD, Tat Chee Avenue, Kowloon, Hong Kong SAR. E-mail: kiwonban@cityu.edu.hk; Hun-Jun Park, MD, PhD, Seoul St. Mary’s Hospital, The Catholic University of Korea, 222 Banpo-daero, Seocho-gu, Seoul, Korea. Email: cardioman@catholic.ac.kr; or Sung-Hwan Moon, PhD, Research Institute, T&R Biofab Co. Ltd, Siheung, Korea. E-mail: [safe33msh@gmail.com](mailto:safe33msh@gmail.com)

**Supplementary Methods**

**Supplementary Methods**

***Ethics approval***

The use of hESCs was approved by the Institutional Review Board of Konkuk University and T&R Biofab Co., Ltd. Animal studies were approved by the Institutional Animal Care and Use Committee and the Department of Laboratory Animals at the Catholic University of Korea (Approval number: CUMC-2020-0051-01). All animal procedures conformed to the guidelines from Directive 2010/63/EU of the European Parliament on the protection of animals used for scientific purposes or the NIH guidelines.

***Human PSC culture and cardiomyocyte differentiation***

The hESC (NKX2.5 **^eGFP/w^** hESC^1^ obtained from Prof. David A. Elliott., and H9) and human induced pluripotent stem cells (hiPSCs; CMC-011^2^) were provided by the Korea National Institute of Health, and TnR-hiPS-4 cells were maintained in iPS-BREW XF medium (StemMACS TM, Mitenyi Biotec, CA, USA) on Matrigel (hESC qualified, Corning, Lowell, MA, USA). For the initial induction of cardiomyocytes, defined small molecules such as CHIR99021 and Wnt-C59 were sequentially treated at 2-day intervals^3, 4^. The cells were then cultured with cardiomyocyte differentiation medium (RPMI1640; Thermo Fisher Scientific, Waltham, MA, USA) + 500 μg/ml human serum albumin (Sigma Aldrich, St. Louis, MI, USA) + 213 μg/ml ascorbic acid (Sigma Aldrich)^5^ for 4 days to generate contractile cardiomyocytes (Figure 1A). All live images were taken with a Lumascope 720 microscope (Etaluma, Carlsbad, CA, USA).

***Manual isolation of NKX2-5 expressing cardiomyocytes***

Differentiated cardiomyocytes were dissociated into single cells using Accutase (Gibco, Amarillo, TX, USA), followed by washing with phosphate-buffered saline (PBS). The dissociated cells were then suspended in PBS containing 5% fetal bovine serum (FBS, Cytiva, Marlborough, MA, USA). Green fluorescent protein positive (GFP^+^) expressing cells were purified using a SH800S Cell Sorter flow cytometer with Cell sorter software Ver 2.1.2 (Sony Biotechnology, San Jose, CA, USA). The sorted cells were identified by fluorescence microscopy, and only GFP^+^ cells were isolated using the mouth pipette technique. Following stable attachment and contraction on culture plates, contractile and non-contractile cells were manually separated using a Pasteur glass pipette (Corning).

***cDNA synthesis of ultra-low input RNA for Illumina sequencing protocols***

The RNA isolated from each sample was used to construct sequencing libraries with the SMART-Seq® v4 Ultra® Low Input RNA Kit for Illumina, following the manufacturer's protocol. First-strand cDNA synthesis was primed by the 3′ SMART-Seq CDS Primer II A, and we used the SMART-Seq v4 oligonucleotide for template switching at the 5′ end of the transcript. The first-strand cDNA selectively binds to SPRI beads, leaving contaminants in the solution, which were removed by magnetic separation. The beads were then directly used for PCR amplification. Advantage 2 Polymerase Mix has been specially formulated for efficient and accurate amplification of cDNA templates by long-distance PCR. PCR-amplified cDNA was purified by immobilization on AMPure XP beads. The beads were then washed with 80% ethanol, and cDNA was eluted with the elution buffer. The Covaris AFA system was used for controlled DNA shearing prior to generating the final library for Illumina sequencing. The resulting DNA was in the 200–400 bp size range. These cDNA fragments then went through an end repair process, the addition of a single ‘A’ base, and then ligation of the indexing adapters. The products were then purified and enriched by PCR to create the final cDNA library. The libraries were quantified using qPCR according to the qPCR Quantification Protocol Guide (KAPA Library Quantification kits for Illumina Sequencing platforms) and qualified using the Agilent Technologies 4200 TapeStation (Agilent Technologies, Waldbronn, Germany). Indexed libraries were then submitted to an Illumina HiSeq 2500 system (Illumina, Inc., San Diego, CA, USA), and paired-end (2 × 100 bp) sequencing was performed by Macrogen Inc.

***mRNA-Seq data***

We processed reads from the sequencer and aligned them with Homo sapiens (hg19) using TopHat v2.0.13^6^. TopHat incorporates the Bowtie v2.2.3 algorithm to perform alignment^7^. TopHat initially removes a portion of the reads based on the quality information accompanying each read before mapping reads to the reference genome. The reference genome sequence of Homo sapiens (hg19) and annotation data were downloaded from the UCSC table browser (http://genome.uscs.edu). Gene annotation information was also used for running TopHat with “-G” option. In case of other parameters for TopHat, the default options were used. TopHat allows multiple alignments per read (up to 20 by default) and a maximum of two mismatches when mapping the reads to the reference. Transcript assembly and abundance estimation were done using Cufflinks^8^. After aligning reads with genome Cufflinks v2.2.1, the aligned reads were assembled into transcripts and their abundance was estimated. To correct the sequence expression count bias, ‘--max-bundle-frags 50000000’ options were used. We also used the ‘-G’ option to make the best use of known gene annotation information. For other parameters, the default options were used. The transcript counts at the isoform level were calculated, and the relative transcript abundances were measured in fragments per kilobase of exon per million fragments mapped (FPKM) from Cufflinks. Gene expression values were also calculated from transcript counts. These values were later used for the differentially expressed gene (DEG) analysis.

***Statistical analysis of gene expression level***

The transcript-level relative transcript abundances were measured in FPKM using Cufflinks. Gene-level relative abundances were calculated as the sum of the FPKMs of transcripts in the gene. We performed statistical analyses to identify DEGs. Genes with one more than zero FPKM values in the samples were excluded. To facilitate log2 transformation, 1 was added to each FPKM value of the filtered genes. The filtered gene data were log2-transformed and subjected to quantile normalization. Statistical significance of the differential expression data was determined using an independent t-test and fold change, in which the null hypothesis was that no difference exists among groups. The false discovery rate (FDR) was controlled by adjusting the p value using the Benjamini–Hochberg algorithm. For the DEG set, hierarchical clustering analysis was performed using complete linkage and Euclidean distance as measures of similarity. Protein-protein interaction network analysis for cell surface related genes was performed using STRING (string-db.org/, Ver.11.0). All data analyses and visualization of DEGs were conducted using R 3.1.2 (www.r-project.org).

***Immunocytochemistry***

Cells were fixed with 4% paraformaldehyde for 20 min and permeabilized with 0.1% Triton X-100 in PBS (Sigma Aldrich) for 5 min. After treatment with 5% normal goat serum for 30 min, the cells were stained with cardiac troponin T (cTnT, Abcam, Cambridge, UK) and α-actinin (Sigma Aldrich) for 12 h at 4 °C. Cells were washed three times with PBS and then incubated with Alexa Fluor 488-or 555-conjugated secondary antibodies (Thermo Fisher Scientific) for 1 h. Nuclei were stained with DAPI (Thermo Fisher Scientific). All images were analyzed using a fluorescence microscope (TE2000-U, Nikon, Tokyo, Japan).

***Electrophysiology***

Current clamp experiments were carried out using a conventional whole-cell configuration for action potential recording^9^. Single cardiomyocytes or aggregates were perfused at physiological temperature (37 °C) with normal Tyrode’s solution containing 145 mM NaCl, 4.3 mM KCl, 1 mM MgCl_2_, 1.8 mM CaCl_2_, 10 mM HEPES, and 5 mM glucose, adjusted to pH 7.4, with NaOH. The intracellular pipette solution contained 120 mM K-aspartate, 20 mM KCl, 10 mM HEPES, 0.1 mM EGTA, 1 mM MgCl_2_, 3 mM Mg-ATP, adjusted to pH 7.25 with KOH. Micro glass patch pipettes (World Precision Instruments, Sarasota, FL, USA) were pulled using a PP-830 puller (Narishige, Tokyo, Japan) with resistance between 2.5–3.0 MOhm. An Axopatch 200 B amplifier, Digidata 1440A, and pClamp software 10.1 (Axon Instruments, Foster, CA, USA) were used for recording and analysis.

***Flow cytometry analysis and cell sorting***

Cells were suspended in PBS containing 1% FBS and incubated with PE-Cy7-conjugated mouse anti-human SIRPα (BioLegend, San Diego, CA, USA) and PE-conjugated mouse anti-human CD71 (BD Biosciences, Franklin Lakes, New Jersey, USA). Cell sorting was performed using the SH800S Cell Sorter flow cytometer with Cell Sorter software Ver 2.1.5 (Sony Biotechnology). For intracellular flow cytometry, cells were harvested with TrypLE Select (Gibco), fixed in 4 % paraformaldehyde for 15 min at room temperature (25–28 °C), blocked, and permeabilized in block buffer consisting of 1 × Perm/Wash Buffer (BD) and 5% goat serum (Sigma Aldrich) for 15 min at 4 °C. Cells were then incubated with cTnT antibody (Abcam) for 1 hat 4 °C, and then treated with Alexa Fluor 488 conjugated secondary antibody (Thermo Fisher Scientific) for 1 h at 4 °C. Flow cytometric data were collected using a SONY SH800S flow cytometer with the analyzed software Ver 2.1.2.

***Formation of aggregate cardiomyocytes and their cryopreservation***

We used two commercial products to form cell aggregates. Purified cardiomyocytes (1 × 10^5^ cells/ml) were transferred into ultra-low attachment (ULA) culture plates (Corning) or a spheroid forming dish (SFD) (SPL Life Science, Pocheon-si, South Korea), and cultured in 10 ml low glucose DMEM (Gibco) medium containing 5% FBS (Gibco) for 1 d in the suspension. In the ULA plate, random agglomerates are formed, whereas in the SFD, uniform aggregates are formed. The size of the aggregates by diameter was measured using the Image J, and the average diameter of the cardiomyocytes aggregates were divided into 100 µm or more and 100 µm or less, and the average was obtained (*n*=10)

For cryopreservation, aggregates were gently washed with PBS, resuspended in culture medium, centrifuged, and allowed to settle by gravity^10^, and the supernatant was removed. Aggregates were mixed with CryoStor (CS10; Biolife Solution, Hong Kong, China) in Cryo-vials (Cryotube; Thermo Fisher Scientific) and cooled to −30 ℃ for 1 h. The vials were then transferred to a liquid nitrogen tank (MVE-XC-47/11; Chart-MVE) and cryopreserved for 6 months. For thawing, the frozen vial was warmed in a 37 °C bead-bath for 1 min, resuspended in 10 ml low glucose DMEM (Gibco) medium containing 5% FBS (Gibco), and centrifuged at 1000 rpm for 3 min. After removing the supernatant, it was washed twice with saline and the prepared aggregates were then used for analysis and animal experiments.

***Analysis of cell viability in hypoxic conditions***

To analyze cell viability under hypoxic culture conditions, single and aggregated cells were cultured in suspension in a 3% O_2_ incubator (Biofree, Seoul, South Korea) for 3 days. The cultured cells were stained with live/dead staining solution (LIVE/DEAD viability kit, Invitrogen, Waltham, MA, USA) according to the manufacturer’s instructions. In brief, 10 ml of DMEM (Gibco) containing 2 μl of calcein AM solution (LIVE) and 10 μl of ethidium homodimer-1 solution (DEAD) was added to the cell culture plate and incubated at 37 °C for 30 min. For each sample, 25 fields of view were randomly selected (*n*=10) and evaluated to calculate the total number of living (green stain) and dead (red stain) cells^11^.Fluorescence images were obtained using a fluorescence microscope (Nikon ECLIS Ti2-U, Nikon, Japan).

***Myocardial infarction and cell transplantation***

Fisher 344 rats (160–180 g, 8-week-old male, Koatec, Pyeongtaek, South Korea) were anesthetized with 2% inhaled isoflurane and intubated via the trachea with an 18 gauge (G) intravenous catheter. The rats were ventilated with a rodent respirator (55-7058, Harvard Apparatus, Holliston, MA, USA). The body temperature of the rats was maintained with a 37 °C heating pad throughout the operation. The chest was shaved and sterilized with 70% alcohol. MI was induced by tying a suture with sterile polyethylene glycol tubing (22G) placed into the left anterior descending (LAD) artery for 1 min, and then the knot was permanently ligated using a 7-0 Prolene suture. Immediately after left coronary artery occlusion, microcardiac spheroids and single cells were injected at two different sites in the border zone of the infarcted myocardium. The rats were randomly divided into the following groups (n≥6 in each group): 1) MI control with PBS, 2) single cells (1 x10^6^), and 3) aggregates (2.4 x 10^4^; formed by using an equivalent number of cardiomyocytes 1 × 10^6^). For histological studies, cells were labeled with chloromethylbenzamido (CellTracker CM-DiI, ThermoFisher, Amarillo, Texas, USA) to trace the injected cells within the heart tissues before transplantation. The chest was closed aseptically followed by administration of antibiotics and 0.9% normal saline solution. All rats were immunosuppressed following three standard drugs according to previous reports (azathioprine, 2 mg/kg; cyclosporine A, 5 mg/kg; methylprednisolone, 5 mg/kg) daily^12^.

***Echocardiography***

The animals were lightly anesthetized with isoflurane and maintained at 37 °C using a heating pad. Serial echocardiography was performed at 1, 2, 4, and 8 weeks after the treatment using a transthoracic echocardiography system equipped with a 15 MHz L15-7io linear transducer (Affniti 50G, Philips, Amsterdam, Netherlands) and the ejection fraction (EF), fractional shortening (FS), left ventricular end-diastolic diameter (LVIDd), left ventricular end systolic diameter, related wall thickness (RWT), septal wall thickness (SWT), and posterior wall thickness (PWT). All parameters were measured from the M-mode at the mid-papillary muscle level. The echocardiography operator was blinded to the group allocation during the experiment^12, 13^.

EF (%) = [(LVEDV − LVESV)/LVEDV] × 100

FS (%) = [(LVEDD − LVESD)/LVEDD] × 100

***Hemodynamic measurements***

Hemodynamic measurements were performed at the end point of 8 weeks before euthanasia. After thoracotomy without bleeding, the left ventricular (LV) apex of the heart was punctured with a 26-G needle, and a 2F conductance catheter (SPR-838, Millar) was inserted into the LV. LV pressure-volume (PV) parameters were continually recorded using a PV conductance system (MPVS Ultra, emka TECHNOLOGIES, Paris, France) coupled to a digital converter (PowerLab 16/35, ADInstruments, Colorado Springs, CO, USA). Load-independent measurements of cardiac function, including the slopes of end-systolic pressure volume relationship (ESPVR) and end-diastolic pressure volume relationship (EDPVR), were performed with different preloads. These were elicited by transient inferior vena cava (IVC) occlusion with a needle holder. Fifty microliters of hypertonic saline (20% NaCl) was injected into the left jugular vein to calculate the parallel conductance after hemodynamic measurements. Blood was collected from the left ventricle into a heparinized syringe and placed into cuvettes to convert the conductance signal to volume using a catheter. The absolute volume of the rat was defined by calibrating the parallel conductance and cuvette conductance ^25-27^.

***Histological analysis***

Eight weeks after the injection of DiI-labeled hiPS-CM, rats were euthanized, perfused with normal saline, and perfusion-fixed with 4% PFA. The heart tissue was removed, fixed in 4% PFA at 4 °C overnight, and then incubated with 30% sucrose solution until it sank. Frozen sections of the heart tissue were obtained at 8 µm thickness. For assessment of fibrosis, Masson’s trichrome staining (Sigma Aldrich) was applied to quantify the percentage of fibrosis and viable myocardium in the left ventricle according to the manufacturer’s instructions. Three different levels from the apex to the top were used for the quantification.

***Immunohistochemistry***

For immunohistochemical analysis, frozen heart tissues were washed with PBS, permeabilized with PBS containing 0.5% Triton X-100 for 15 min and blocked with 3% BSA solution for 1 h at RT. The cells were then incubated with the following primary antibodies at 4 °C overnight: ACTN2 (Sigma Aldrich), cTnT (Thermo Fisher), MYH6/7 (Abcam), hMYH7 (Abcam), CD31 (Abcam), and GJA1 (Abcam). The cardiac tissues were washed three times with PBS and incubated with secondary antibodies for 1 h at RT. The secondary antibodies used in this study included either anti-mouse/-rabbit IgG Alexa Fluor 488 (Invitrogen) or anti-mouse/-rabbit IgG Alexa Fluor 647 (Invitrogen). After three washes with PBS, the slides were mounted with VECTASHIELD mounting medium containing DAPI (Vector Laboratories, Burlingame, CA, USA). Immunohistochemical analysis was performed using three to five independent samples. All *in vitro* images were acquired and analyzed using a Nikon A1R HD25 confocal microscope (Nikon Corp., Tokyo, Japan).

***Statistical analysis***

All quantitative data are shown as mean ± standard error of mean unless otherwise indicated. Statistical differences between two groups were analyzed using two-tailed Student’s t-tests. The statistical differences among the three groups were also analyzed using analysis of variance with Bonferroni’s post-hoc analysis. The results were considered statistically significant when the p-value was less than 0.05.

**Supplementary Figures List**

**Supplementary Figures 1**. Differentiation of human pluripotent stem cells (hPSCs) into cardiomyocytes (CMs)

**Supplementary Figures 2**. Expression of the cell surface protein CD71 during hPSC differentiation**.**

**Supplementary Figures 3**. Fibroblast aggregates in hypoxic conditions and after long-term cryopreservation were characterized using LIVE/DEAD assay.

**Supplementary Figures 4**. Electrophysiological function analysis using patch clamp.

**Supplementary Figures 5**. Transplantation of human induced pluripotent stem cell-derived cardiomyocytes (CMs) and aggregated CMs in myocardial infarction (MI).

**Supplementary Figures 6.** Hemodynamic cardiac function at steady state. (A) Representative image of the hemodynamic pressure and volume of each group

**Supplementary Figures 7**. Hemodynamic cardiac function at steady state

**Supplementary Figures 8**. Intrinsic cardiac function.


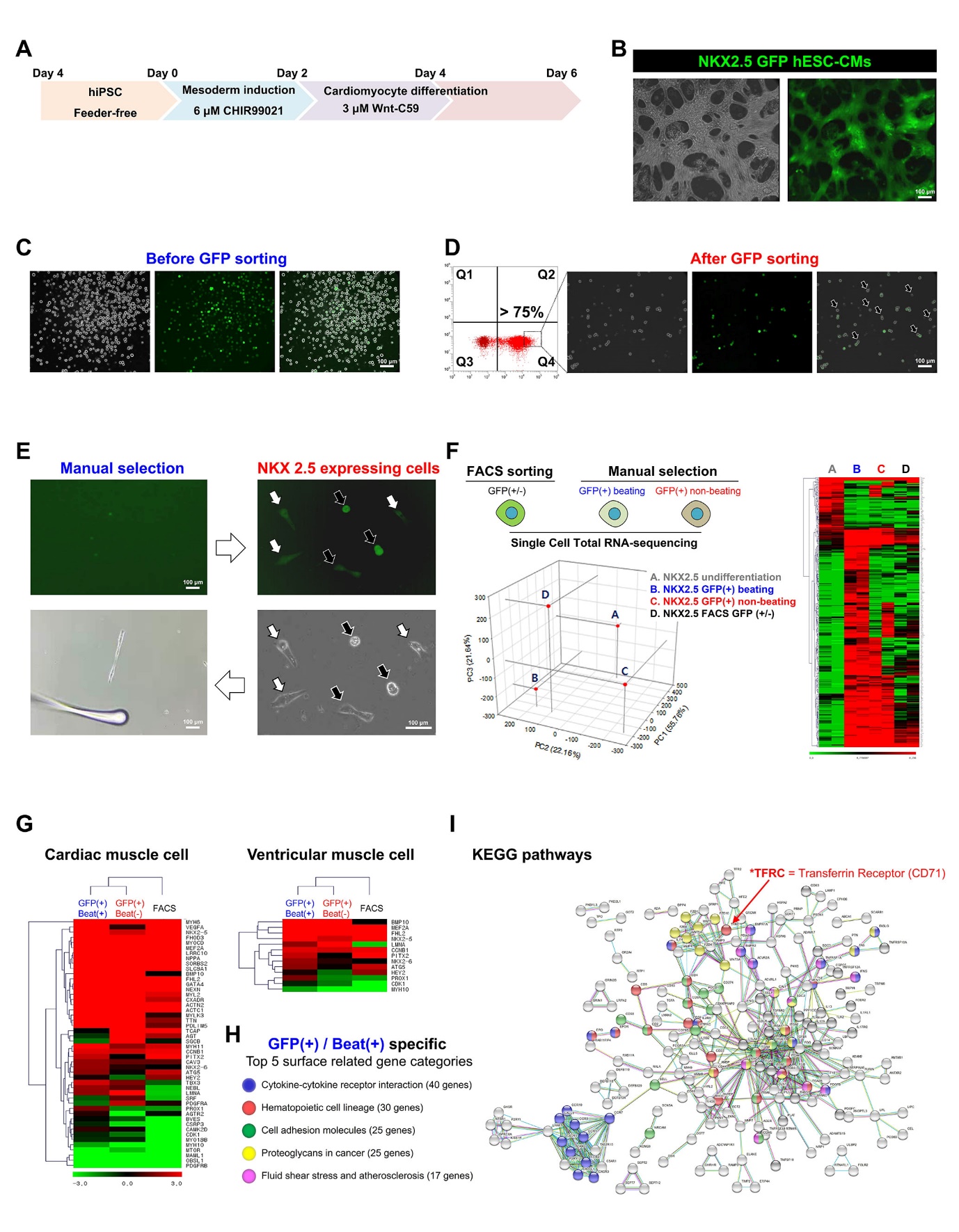


**Supplementary Figure 1. (A) through (B), Differentiation of human pluripotent stem cells (hPSCs) into cardiomyocytes (CMs). (A)** Schematic for cardiomyocyte differentiation of hPSCs. **(B)** Detection of GFP in NKX2.5 ^eGFP/w^ culture at day 8 of cardiac differentiation (Supplementary live image 1). **(C) and (D)** FACS sorted GFP^+^ cardiomyocytes derived from NKX2.5 ^eGFP/w^ hESCs. **(E)through (I), Screening of contracting cardiomyocyte specific surface maker**. (**E)**Manual selection of beating GFP positive cells after FACS sorting. (**F**) Comparison of gene expression profiles between NKX2.5 GFP^+^ beating and non-beating cardiomyocytes. Principal component analysis of each cardiomyocytes using differential expressed genes (DEGs). Heat map of global gene expression in hESC-derived cardiomyocytes (left panel). Heat map of cardiac muscle cell development and ventricular cardia muscle cell development in hESC-derived cardiomyocytes (right panel). (**G and I**) The top 5 most enriched KEGG pathways of 288 up-regulated genes in NKX2.5 GFP^+^ beating cardiomyocytes (fold change >3). The protein-protein interaction network analysis of the 288 cell surface genes. The circle nodes represent genes. Red indicates genes in hematopoietic cell lineage, blue represents genes in cytokine-cytokine receptor interaction, green indicates genes in cell adhesion molecules, yellow represents genes in proteoglycans in cancer, and pink indicates genes in fluid shear stress and atherosclerosis.


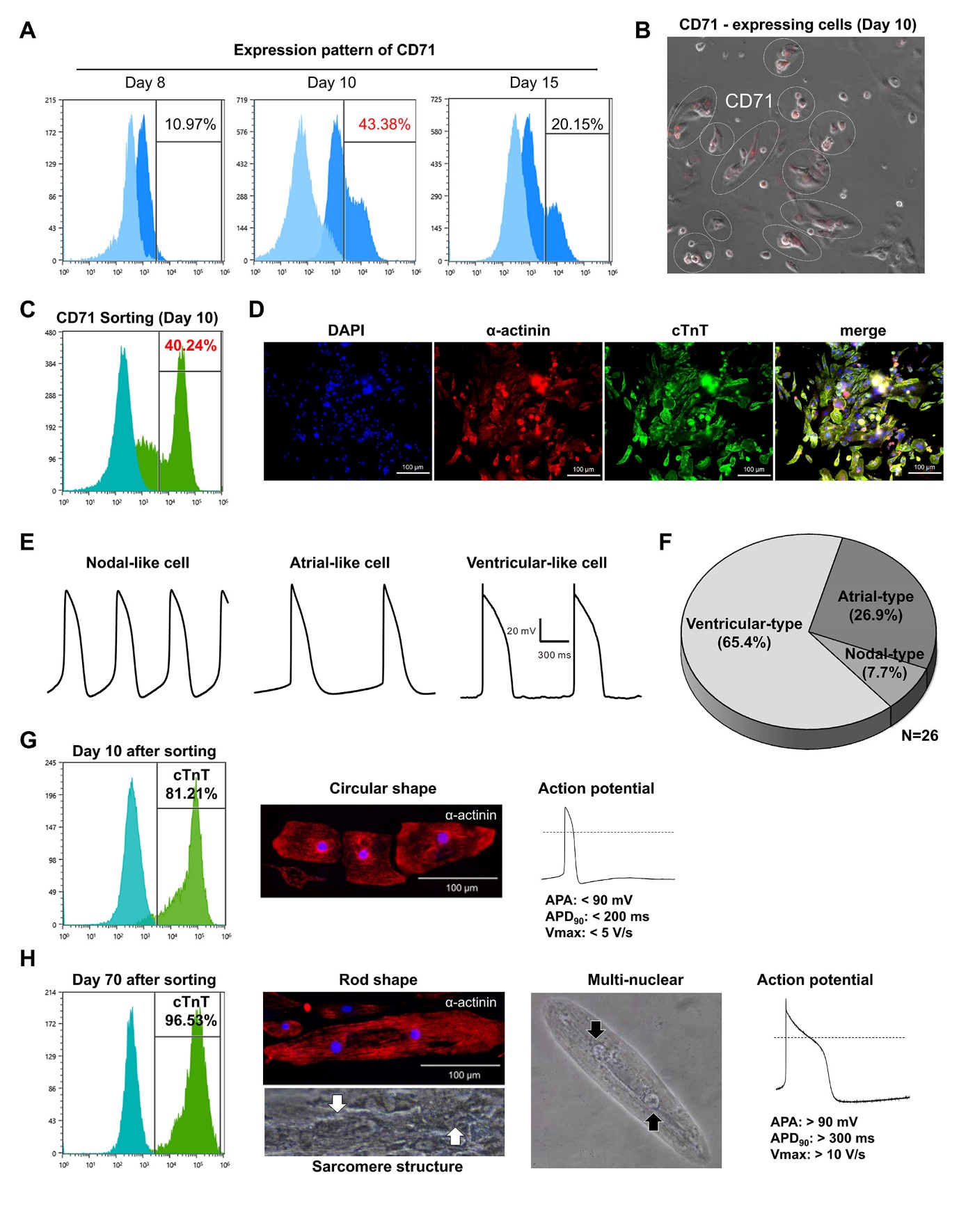


**Supplementary Figure 2. Expression of the cell surface protein CD71 during hPSC differentiation.** (**A)** Flow cytometric analysis of CD71 expression in H9-hESCs derived cardiomyocytes at the indicated times. (**B)** Detection of CD71 in cardiomyocytes derived H9-hESCs (white circle, Supplementary live image 5). (**C)** FACS sorted CD71^+^ cardiomyocytes**.** (**D)** Immunostaining for cTnT, α-actinin on a monolayer culture generated from CD71+ cells sorted on day 10. Scale bar, 100 µm. **(E)** and **(F)** Electrophysiological analysis; dotted lines indicate 0 mV and single action potentials were obtained from the region indicated with an asterisk. Ratio of the three major types of action potentials in CD71^+^ cardiomyocytes. **(G)** CD71^+^ CMs (left panel) morphology, α-actinin expression (middle panel) and action potential (right panel) 10 days after sorting. (**H)** CD71^+^ CMs (left panel) morphology, α-actinin expression (middle panel), and action potential (right panel) 70 days after sorting.


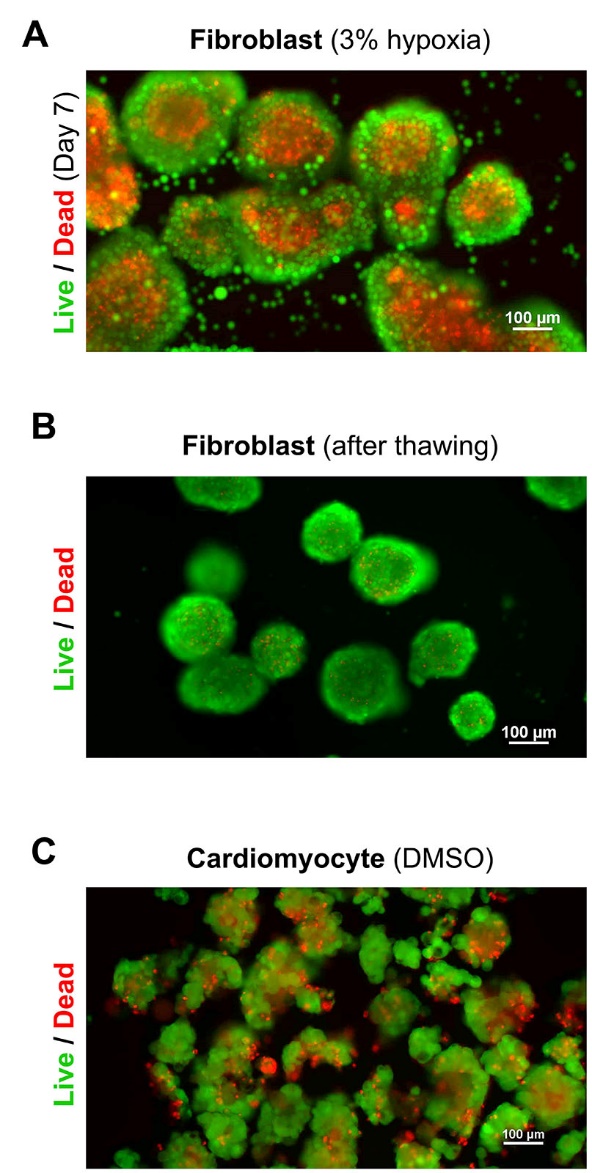


**Supplementary Figure 3.** Fibroblast aggregates in hypoxic conditions and after long-term cryopreservation were characterized using LIVE/DEAD assay. (A) The viability of fibroblast aggregates was analyzed using LIVE/DEAD assay in 3 % hypoxia condition. (B) Survival analysis of ≤300 µm fibroblast aggregates after thawing. (C) Viability of fibroblast aggregates after cryopreservation using DMSO-supplemented freezing medium.


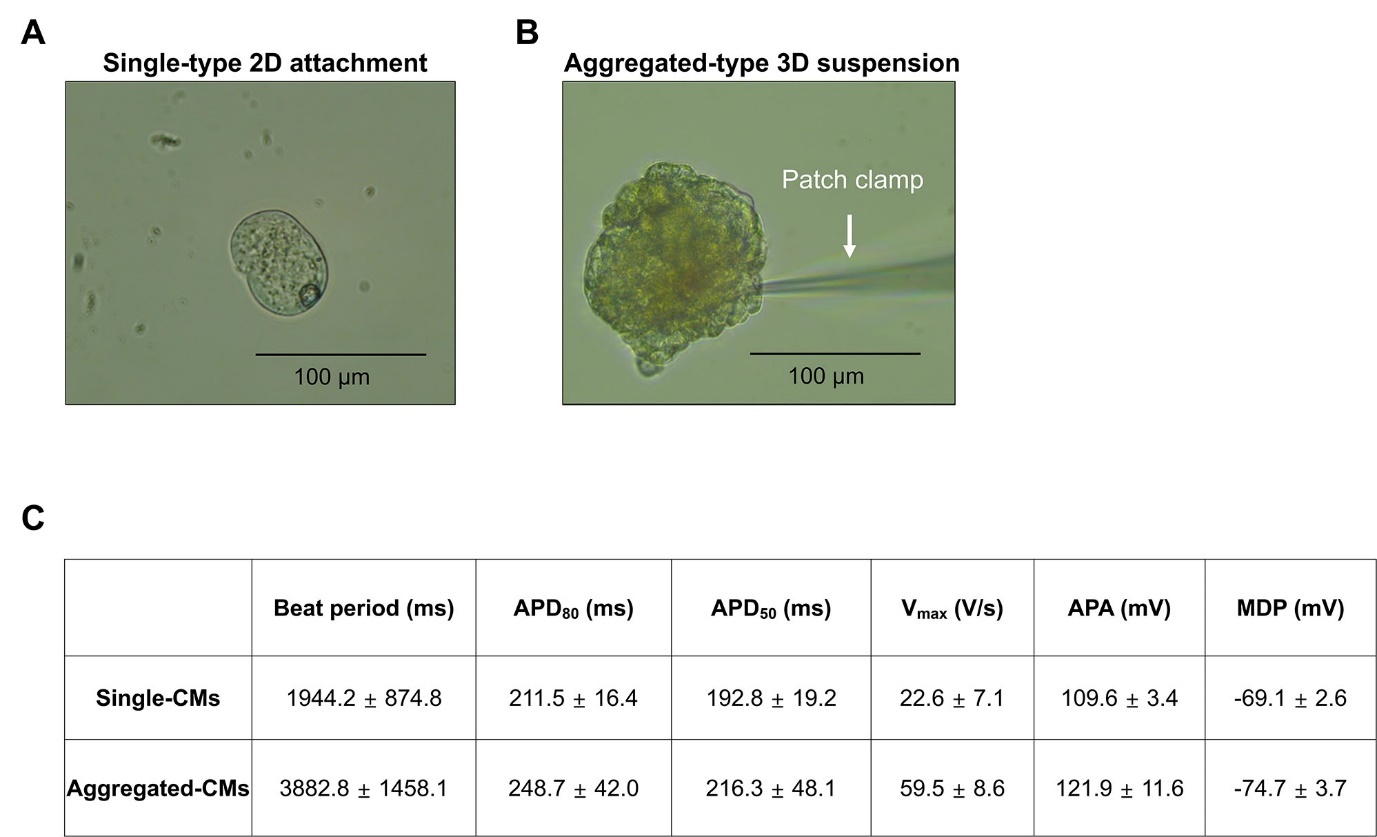


**Supplementary Figures 4**. Electrophysiological function analysis using patch clamp. (A) and (B) Representative image of single and aggregated cardiomyocyte for patch clamp analysis. (C) Summary of action potential (AP) parameters obtained from single-cardiomyocytes (n=17) and aggregated cardiomyocytes (n=22). AP parameters (mean ± S.D.) were determined by averaging data from ventricular-type APs at steady state (APD80 and APD50: action potential duration at 80 % and 50 % of repolarization; Vmax: maximum upstroke velocity; APA: action potential amplitude; MDP: maximum diastolic potential).


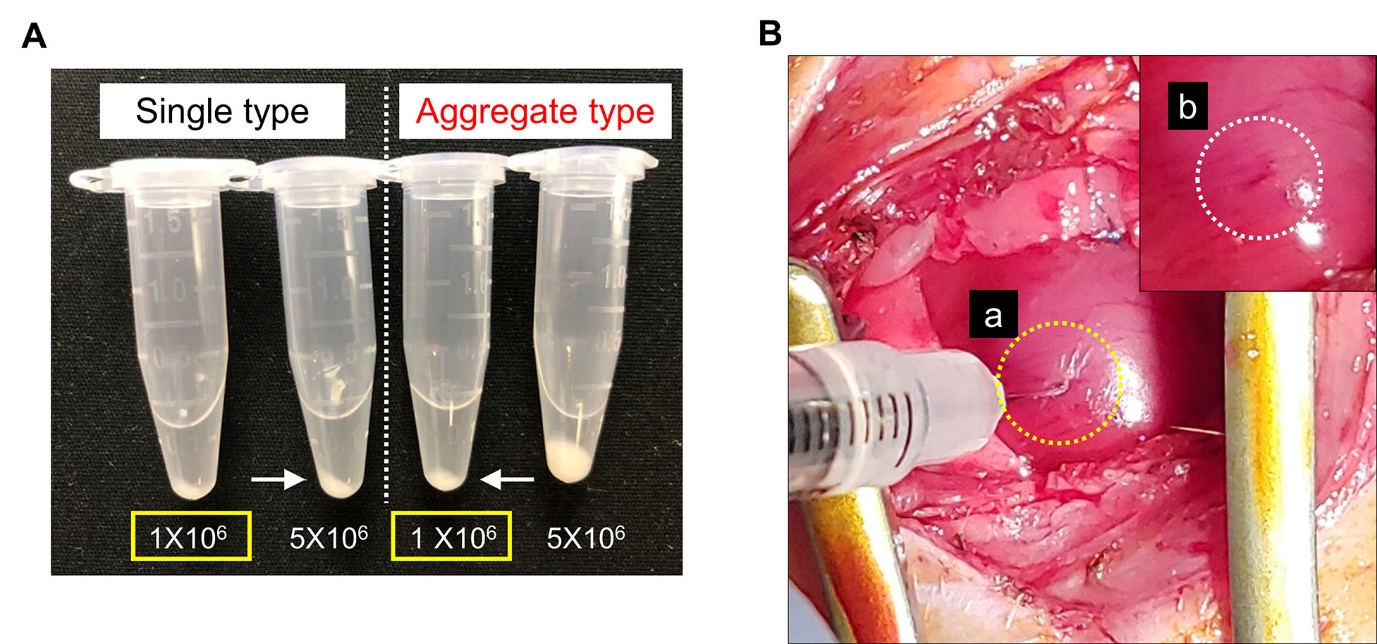


**Supplementary Figure 5.** Transplantation of human induced pluripotent stem cell-derived cardiomyocytes (CMs) and aggregated CMs in myocardial infarction (MI). (A) Comparison of volume by cell number of single type and aggregate type. The experimental groups were divided into two volumetrically equivalent MI groups: 1 × 10^6^ single cardiomyocytes and 2.4 × 10^4^ aggregates CMs (equivalent cell number of single cells ;yellow box; cell numbers, white arrows; cell pellet volume) (B) The aggregates and single-type cardiomyocytes were injected at two different sites in the border zone of the infarcted myocardium after MI. (a) before injection: border zone (b) after injected: There were no signs of hemorrhage or blood loss during the entire procedure.


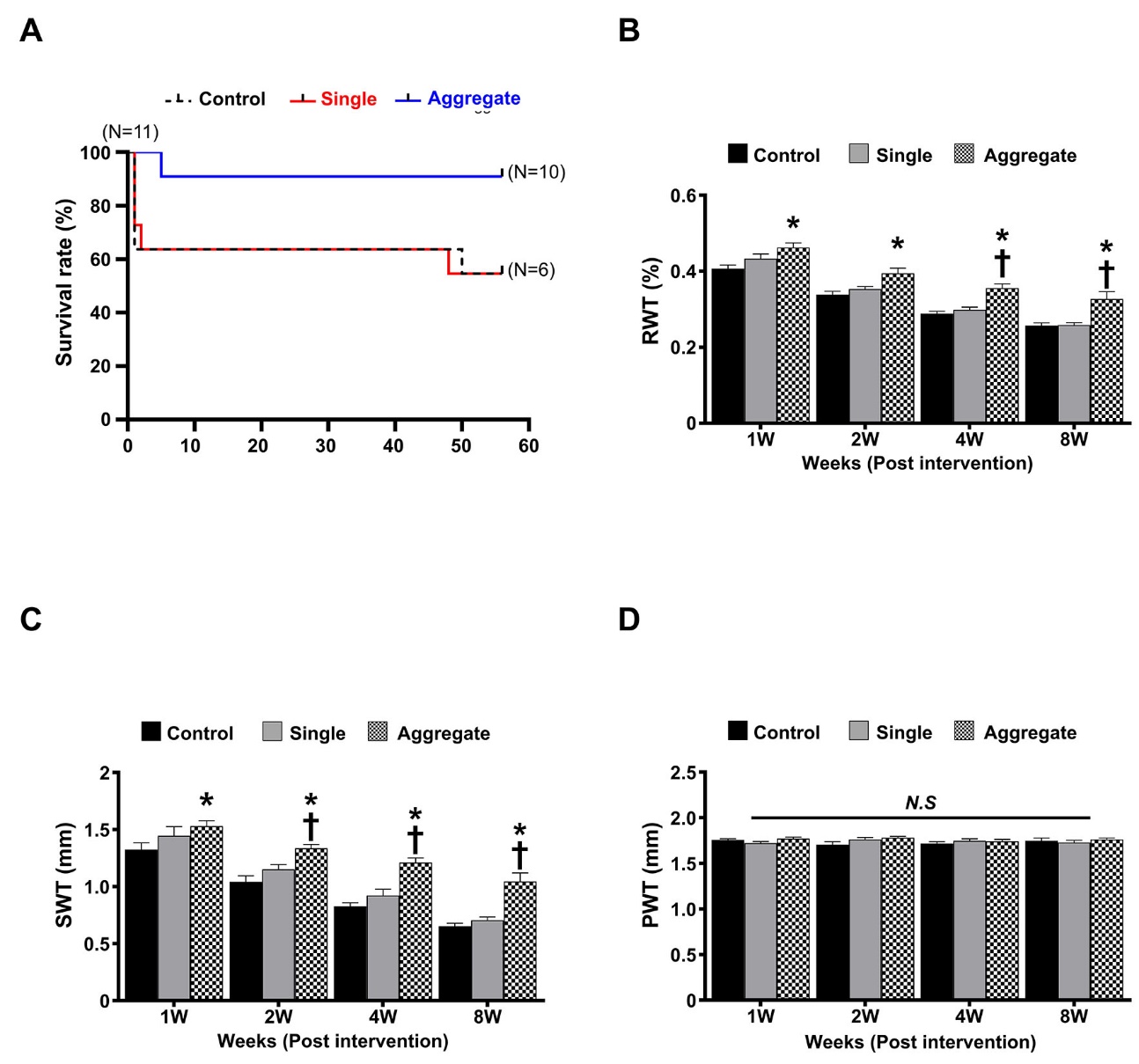


**Supplementary Figure 6.** Kaplan-Meier curve analysis and echocardiography. (A) Kaplan-Meier curve over 8 weeks after transplantation. (B) Related wall thickness. (C) Septal wall thickness. (D) Posterior wall thickness. Data are expressed as the mean ± standard error of mean (SEM). *p<0.05 compared to control group; †p<0.05 compared to the single CM-treated group. Two-way analysis of variance (ANOVA) was followed by multiple comparisons with the Tukey method (n=6 to 10 per experimental group).


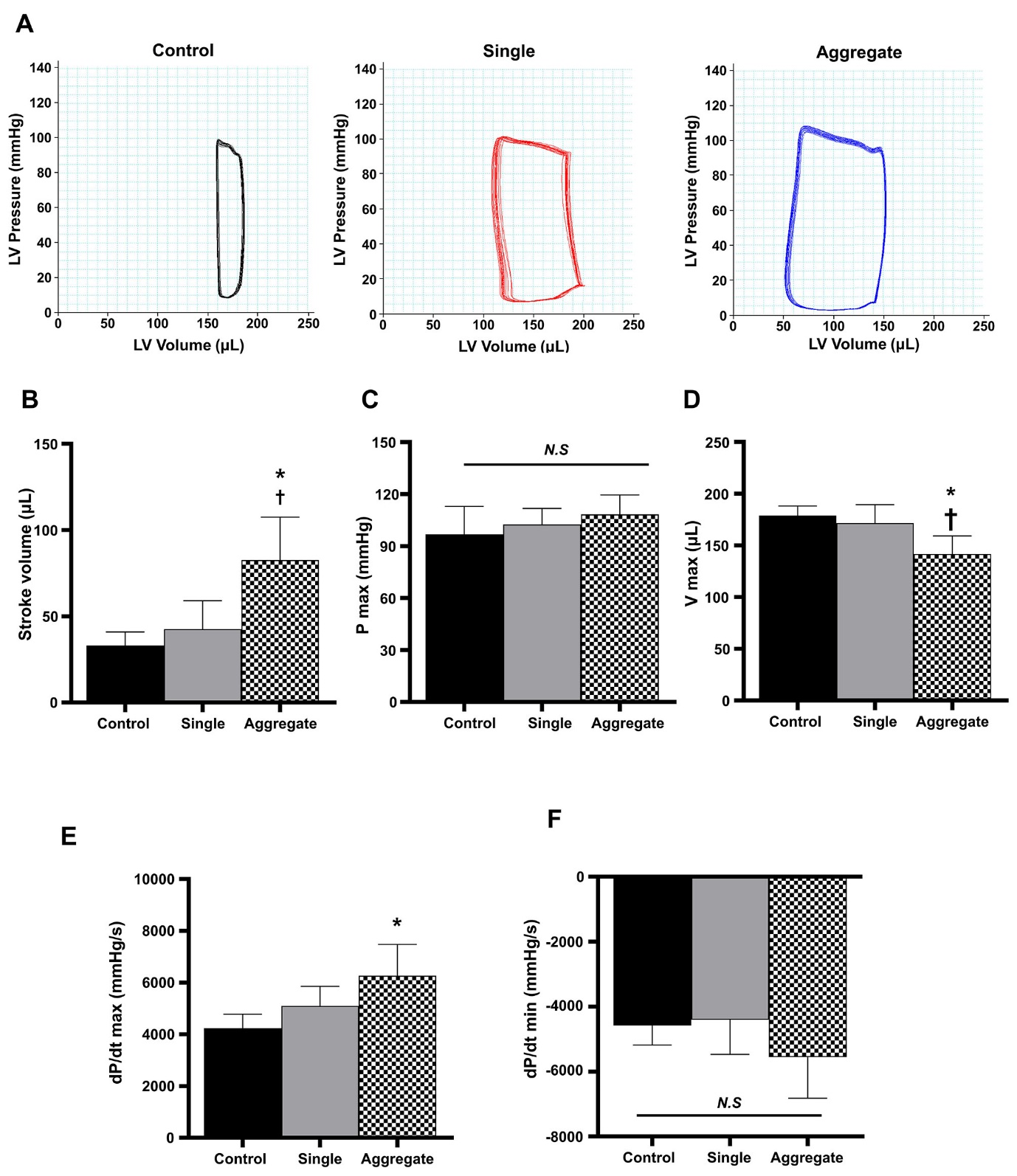


**Supplementary Figure 7.** Hemodynamic cardiac function at steady state. (A) Representative image of the hemodynamic pressure and volume of each group. (**B**) stroke volume (SV). (**C)** Pressure max (P max) at the maximum systole**. (D)** Cardiac remodeling as measured by the volume max (V max**). (E)** Systolic cardiac function as measured by the maximal rate of pressure change during systole (dP/dt_max_). **(F)** Minimal rate of pressure changes during diastole (dP/dtmin). Data are expressed as the mean ± SEM. One-way ANOVA was followed by multiple comparisons with the Tukey method (n=5 to 6 per experimental group).


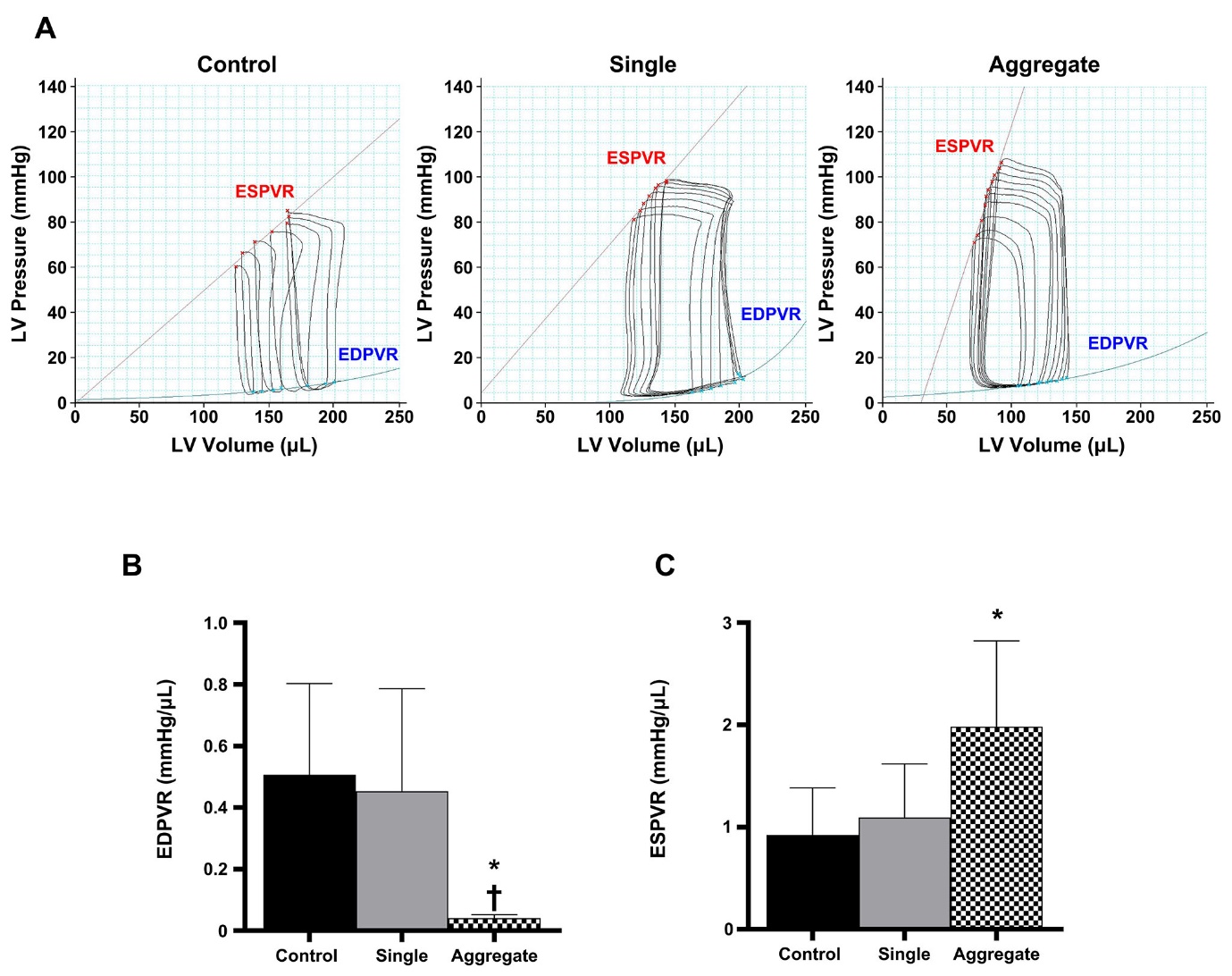


**Supplementary Figure 8.** Intrinsic cardiac function. **(A)** Representative image of cardiac function load-independently each groups. **(B)** Slope of end-systolic pressure volume relationship (ESPVR). **(C)** Slope of end-systolic pressure volume relationship (ESPVR) by transient Inferior vena cava (IVC) occlusion. Data are expressed as the mean ± SEM. One-way ANOVA was followed by multiple comparisons with the Tukey method (n=5 to 6 per experimental group).**SUPPLEMENTARY NOTES**

**1. Screening of surface markers relevant to contractile cardiomyocytes**

Single-cell total RNA sequencing analysis was performed to construct gene expression profiles of manually isolated cells and FACS-sorted cells for the identification of cardiomyocyte-specific surface markers. RNA was extracted from four distinct groups (undifferentiated, GFP+/beating, GFP+/non-beating, and FACS-sorted GFP+/− cardiomyocytes) to quantify and compare the expression levels of relevant genes at the transcription level. Principal component analysis revealed that the three types of isolated cells were distinctly distinguishable from the undifferentiated cells and exhibited characteristics similar to those of cardiomyocytes (Figure S1E). A heat map comparison also showed that NKX2.5 GFP+ contracting cells displayed a unique expression pattern that contrasted with that of the other cardiomyocyte groups (Figure S1F;left). Our analysis showed that cardiac muscle and ventricular muscle cell-specific genes were highly upregulated, suggesting that the isolated cardiomyocytes may be ventricular in nature (Figure S1F; right). To screen for an appropriate surface marker for GFP+ contracting cardiomyocytes, specific genes (GO:0009986) were first identified, and 288 upregulated cell surface genes (>3-FC) were selected before performing the KEGG pathway annotation using STRING (http://string-db.org/). The five most enriched KEGG pathways of the upregulated genes with an FDR of <0.01 and an interaction score of high confidence (>0.7) included the hematopoietic cell lineage, cytokine-cytokine receptor interaction, cell adhesion molecules (CAMs), proteoglycans in cancer, and fluid shear stress and atherosclerosis (Figure S1G). The network of the 288 cell surface genes, with 286 nodes and 666 edges, is presented as the results of the KEGG pathway analysis (Figure S1H and I, Supplementary Table 1). Of the 288 surface marker-related genes identified, the transferrin receptor (TRFC, CD71) gene, which is involved in left ventricular remodeling, was selected as a potential candidate because of the well-known induction of lethal cardiomyopathy in CD71/TRFC-knockout mouse models^14^.

**2. Optimization of cryopreservable aggregates with improved survival in hypoxia**

The limitation of therapeutic efficacy due to the poor *in vivo* survival rate of transplanted cells is an important challenge to overcome for stem cell therapy^15^. Therefore, techniques that improve the survival of transplanted cardiomyocytes are urgently needed. Although various methods for improved survival, such as cytokines, materials, and 3D printing, are being developed^12, 16, 17^, regulatory hurdles for clinical applications remain to be addressed. From a clinical point of view, cell aggregation by physical methods has been proposed, and we previously reported that aggregation improved cardiomyocyte survivability and engraftment rates in rat MI models^11^. However, the underlying mechanism through which aggregation improves *in vivo* survival is still unclear, and the optimal aggregate size for encouraging this phenomenon is unknown. In this study, we hypothesized that aggregates remain viable under hypoxic conditions, which are known to occur in the physiological microenvironment inside the human body^18^. Under 3% hypoxic conditions, dissociated cardiomyocytes were cultivated on gelatin-coated or ULA dishes for 3 days, and cell survival was confirmed by live/dead staining (Figure 1D). On day 1, most cells survived either as adherent monolayers on gelatin or as aggregates on ULA. However, > 90% of the adherent cells died by day 3, whereas > 50% of the aggregated cardiomyocytes were alive (Figure 1E). Based on these observations, cardiomyocytes appear to undergo spontaneous aggregation in the absence of adhesion and proliferation to survive hypoxic conditions. Notably, the death rate of large aggregates (>300 μm in diameter) formed on day 1 was high on day 3 (Figure 1D; white arrows) but aggregates below 200 μm in diameter (Figure 1D; yellow bar) remained viable for more than 7 days (data not shown). To determine whether long-term survival was dependent on non-proliferation, aggregates were formed with fibroblasts under hypoxic conditions due to their known proliferative properties. As expected, cell death was observed inside the aggregates as it grew in size due to the proliferation of cells (Figure S3A). Taken together, these results suggest that cell aggregates below 200 μm in diameter are optimal for the long-term survival of non-proliferating cardiomyocytes in a hypoxic environment. In the case of acute MI, where a patient requires immediate treatment, viable aggregates in a cryopreserved state should be readily available. To test aggregate viability after long-term cryopreservation, various sizes (<100 μm, 100–300 μm, and >300 μm) were selected at random, frozen in CryoStor10 cryopreservation medium, and cryopreserved in liquid nitrogen for 6 months (Figure 1F). An analysis of post-thaw survival revealed that 80% of the surviving aggregates were below 300 μm in diameter and larger aggregates displayed a significant reduction in survival rate by approximately 20% (Figure 1F and 1G, supplementary live images 10 and 11). Similar observations were made with proliferative fibroblasts when the same experiment was performed after 6 months of cryopreservation (Figure S3B). Long-term survival was dependent on cell proliferation capacity, as aggregate size below 300 μm was optimal for both fibroblasts and cardiomyocytes. In tandem, selection of the cryopreservation medium is also an important factor. In DMSO-containing freezing medium, the survival of aggregates (≤300 μm in diameter) was reduced (Figure S3C). These results suggest that aggregation not only improves survival in a hypoxic environment but also protects cells against physical stress during freezing. A technique capable of forming standardized and uniform aggregates is required to exert consistent therapeutic effects, especially in clinical settings. For uniform aggregates, we considered a diameter less than 200 μm as the optimal aggregate size, which, based on our observations, was able to survive both in hypoxic conditions and after long-term cryopreservation. The aggregates continued to contract for more than 30 days and survived after long-term cryopreservation (≥ 80% survival rate) while retaining electrophysiological functions (Figure 1H, supplementary live images 12 and 13). Interestingly, there was an increase in the proportion of ventricular cells in aggregated cardiomyocytes (≥ 73%) (Figure 1H) compared to single type cardiomyocytes (≥65%) (Figure S2F). Taken together, this approach demonstrates a way to overcome clinical concerns regarding the survival of clinical iPSC-CMs in hypoxia and long-term storage. Previous studies have reported that a cell spheroid of up to 118±32 μm can survive under hypoxic conditions. This was based on the measurement of the oxygen gradient of the spheroid using electron parametric resonance oximetry^19^. After testing various cell aggregation products, such as Concave (StemFIT 3D, Microfit, Hanam, South Korea)^20^ and Aggre-well (Greiner Bio-one, Kremsmünster, Austria)^21^, we chose the SFD (SPL Life Science) for simplicity and convenience. Using SFD, it was possible to form a homogeneous aggregate 105±5 μm in size by plating 41±3 single cells (Figure 1J, Supplementary live images 16 and 17).

**3. Therapeutic efficacy of cryopreserved aggregates for improving cardiac function in acute myocardial infarction**

The efficacy of cryopreserved CAs was analyzed in a preclinical animal model of MI. After the cells were thawed and prior to animal experimentation, it was confirmed that 100-µm aggregates were able to pass through a 27-G syringe without damage unlike the 200-µm aggregates, which fractured in the process (Figure 1I and supplementary live image 14-15). Therefore, 100-µm aggregates can be applied to the clinical NOGA 27G catheter system for transendocardial cell delivery^22^. The experimental groups were divided into two volumetrically equivalent MI groups: 1 × 10^6^ single cardiomyocytes and 2.4 × 10^4^ aggregates CMs (Figure S5A; yellow box). MI models were established by permanently ligating the LAD artery, and each group was injected at two different sites within the border zone of the infarcted myocardium (Figure S5B; (a) yellow circle). There were no signs of hemorrhage or blood loss during the entire procedure (Figure S5B; (b) white circle). Out of 11 subjects (*n*=11) per group, the control and single groups had six survivors at the end of the 8-week experiment, whereas the aggregate group had 10, showing a lower mortality rate in the Kaplan-Meier curve analysis (Figure S6A).

Echocardiographic analysis performed at 1, 2, 4, and 8 weeks post-transplantation revealed a significant decrease in the LVIDd and systolic dimension (LVISd) in the aggregate group after 8 weeks (Figure 2A) compared to the control and single groups (Figure 2B). In addition, the aggregate group showed an increase in the RWT, indicating a reduction in adverse cardiac remodeling (Figure S6B). The left ventricular ejection fraction and FS are important indicators for the evaluation of cardiac function. They were significantly higher in the aggregate group in comparison to the other groups (Figure 2C and 2D) in addition to an increase in SWT (Figure S6C). No differences were detected in the PWT of the non-infarcted region among the three groups (Figure S6D). Taken together, these results suggest that the aggregated cardiomyocytes were more effective at improving impaired cardiac function than single cardiomyocyte transplantation. Further evaluation was conducted with a PV loop analysis that can load-independently measure the hemodynamic pressure and volume of the LV during systole and diastole by inserting a catheter directly into the LV. At 8 weeks post-MI, the results demonstrated improved cardiac function and prevention of adverse cardiac remodeling in the aggregate group in comparison to the control and single groups (Figure 2E and Figure S7A). Hemodynamic parameters such as cardiac output (CO) and stroke volume (SV), which refer to the volume of blood ejected from the heart for 1 min and during one contraction, were increased in the aggregate group (Figure 2F)

The reduction in adverse cardiac remodeling was indicated by a lower value for maximum volume, which refers to the blood volume in the left ventricle at maximum diastole (Figure S7D). No statistically significant differences in pressure within the left ventricle (maximum pressure) at the maximum systole (Figure S7C) were observed across the groups. However, the aggregate group showed 1.5× higher maximal rate of pressure changes during systole, which was determined by the measurement of dP/dt_max_ and dP/dt_min_. This indicates the change in pressure of the left ventricle per second (Figure S7E-F). Overall, these results suggest that the aggregate group was capable of improving the functionality of an infarcted heart. Moreover, load-independent cardiac function was verified by analyzing the slope of the ESPVR and EDPVR by transient IVC occlusion. A significantly steeper and higher ESPVR but lower EDPVR were displayed by the aggregate group when compared to the other groups (Figure S8). This suggests an increase in load-independent heart contractibility after MI. Taken together, the consistency in the results of echocardiography, hemodynamics, and load-independent cardiac analysis distinctly demonstrates the improvement in cardiac function post-MI in subjects that received microcardiac spheroids.

**Summary of Manuscript**

The none-to-marginal benefit of functional recovery has been reported in numerous clinical trials of adult stem cell-based cardiac therapy^23^. hiPSC-CM is considered the next most promising cell type for the treatment of cardiovascular diseases^24, 25^. However, several major issues such as tumorigenicity, immunogenicity, short storage life, low retention, and engraftment must be resolved before its application. In this study, we demonstrate a comprehensive process capable of tackling several of the aforementioned obstacles. First, we showed that TFR-1 or CD71 was selected because of its well-known involvement in left ventricular remodeling. The ablation of the CD71 gene in knockout mice results in embryonic lethality due to a high degree of cardiomyopathy, suggesting the functional importance of this protein^14, 26^. Further, it has been a useful marker for the identification of erythroid precursors in past studies^27^, and could serve to identify and purify contractile hiPSC-CMs.

We further demonstrated the importance of size uniformity in the long-term cryopreservation of microcardiac spheroids as the group containing aggregates with a 100 μm diameter was the only group that exhibited stability in storage. Under hypoxic conditions, spontaneously aggregated hiPSC-CMs displayed a survival rate 5-fold higher than that of single cardiomyocytes, as cell death was undetectable. This is mainly because of the substantial increase in the conexin43 protein because of the proximal intracellular contact of myocardial cells to maintain the integrity of the sphere as a whole^11^. A recent report on electron parametric resonance oximetry analysis also claimed that 118 ± 32 μm cell spheroids were able to survive hypoxic conditions^19^.

Furthermore, microcardiac spheroid treatment significantly improved the rate of survival, retention, and engraftment as well as ventricular function in ischemic hearts despite the absence of supportive materials such as pro-survival cocktails^28, 29, 30^ and decellularized extracellular matrices^12, 31^, which have been suggested to increase the viability of hiPSC-CMs *in vivo*. However, such approaches will add complexity requiring detailed efforts for technical validation and regulatory clearance. In contrast, the method outlined in this study does not require complementation. Therefore, the standard regulatory barriers are to be expected.

Surprisingly, histological analysis revealed the maturation of microcardiac spheroids *in vivo* in relation to the shape, structure, and specific protein expression (Figure 4D). Maturation was not observed in single cell transplants, suggesting that intracellular contact and subsequent cell-cell communication in aggregates is important for survival and drives the maturation of transplanted cells. This phenomenon was absent in our previous study, most likely due to the size variations of aggregates^11^. It appears that uniformity is also essential to induce maturation, thereby reducing the risk of arrhythmia. Essentially, the 100-μm microcardiac spheroids established in this study can be preserved, stored, and exhibited superior viability under hypoxic conditions and *in vivo* for direct implantation into patients in emergency situations. Furthermore, microcardiac spheroids can be used in a clinical 27G NOGA catheter system without physical damage; thus, it may be appropriate for clinical studies.

**Supplementary Table 1.**

**Screening of surface markers relevant to contractile cardiomyocytes list.**

| **Term ID** | **Term description** | **Observed  gene count** | **False  discovery rate** | **Genes** |
| --- | --- | --- | --- | --- |
| hsa04640 | Hematopoietic cell lineage | 30 | 2.18E-24 | ANPEP,CD14,CD1B,CD2,CD33,CD38,CD4,CD44,CD5,CD59,CD8A,EPO,EPOR,FCER2, FLT3LG,IL1R1,IL2RA,IL6R,IL7R,ITGA1,ITGA2,ITGA2B,ITGA3,ITGA4,ITGA6,ITGAM,ITGB3, MS4A1,TFRC,TNF |
| hsa04060 | Cytokine-cytokine receptor interaction | 40 | 1.39E-22 | ACKR3,ACVR2A,BMPR1A,BMPR2,CCR1,CCR10,CCR2,CCR7,CCR9,CD27,CX3CL1,CXCL9, CXCR3,CXCR5,EPO,EPOR,FAS,FASLG,FLT3LG,IFNG,IL13,IL17A,IL17RB,IL17RC,IL1R1, IL2RA,IL6R,IL7R,PDGFB,PDGFC,TGFB1,TGFBR2,TNF,TNFRSF10A,TNFRSF12A,TNFRSF14, TNFRSF18,TNFRSF1A,TNFSF18,TNFSF4 |
| hsa04514 | Cell adhesion molecules (CAMs) | 25 | 1.99E-15 | CD2,CD226,CD274,CD276,CD4,CD58,CD86,CD8A,CDH5,CTLA4,ITGA4,ITGA6,ITGAM,ITGB1, NLGN1,NRCAM,PDCD1,PTPRC,SDC2,SDC3,SELL,SELP,SPN,TIGIT,VTCN1 |
| hsa05205 | Proteoglycans in cancer | 25 | 1.68E-12 | CAV1,CAV3,CD44,CD63,FAS,FASLG,FZD1,FZD10,FZD4,FZD9,ITGA2,ITGB1,ITGB3,ITGB5, PPP1CC,RHOA,SDC2,TGFB1,THBS1,TLR2,TLR4,TNF,WNT3A,WNT5A,WNT6 |
| hsa05418 | Fluid shear stress and atherosclerosis | 17 | 1.97E-08 | ACVR2A,BMPR1A,BMPR2,CAV1,CAV3,CDH5,IFNG,IL1R1,ITGA2B,ITGB3,PDGFB,PLAT, RHOA,SDC2,TNF,TNFRSF1A,TRPV4 |
| hsa04151 | PI3K-Akt signaling pathway | 26 | 2.50E-08 | EPO,EPOR,FASLG,FGF10,FLT3LG,IL2RA,IL6R,IL7R,ITGA1,ITGA2,ITGA2B,ITGA3,ITGA4, ITGA6,ITGB1,ITGB3,ITGB5,LPAR1,PDGFB,PDGFC,PKN1,PKN2,TGFA,THBS1,TLR2,TLR4 |
| hsa05200 | Pathways in cancer | 31 | 6.74E-08 | EPO,EPOR,FAS,FASLG,FGF10,FLT3LG,FZD1,FZD10,FZD4,FZD9,HHIP,IFNG,IL13,IL2RA, IL6R,IL7R,ITGA2,ITGA2B,ITGA3,ITGA6,ITGB1,LPAR1,PDGFB,RALA,RHOA,TGFA,TGFB1, TGFBR2,WNT3A,WNT5A,WNT6 |
| hsa04810 | Regulation of actin cytoskeleton | 18 | 8.76E-07 | CD14,FGF10,ITGA1,ITGA2,ITGA2B,ITGA3,ITGA4,ITGA6,ITGAM,ITGB1,ITGB3,ITGB5,MYH9, PDGFB,PDGFC,PPP1CC,RHOA,SSH1 |
| hsa04512 | ECM-receptor interaction | 12 | 9.54E-07 | CD44,HMMR,ITGA1,ITGA2,ITGA2B,ITGA3,ITGA4,ITGA6,ITGB1,ITGB3,ITGB5,THBS1 |
| hsa05165 | Human papillomavirus infection | 22 | 1.09E-06 | FAS,FASLG,FZD1,FZD10,FZD4,FZD9,ITGA1,ITGA2,ITGA2B,ITGA3,ITGA4,ITGA6,ITGB1, ITGB3,ITGB5,PSEN1,THBS1,TNF,TNFRSF1A,WNT3A,WNT5A,WNT6 |
| hsa04145 | Phagosome | 15 | 1.27E-06 | CD14,FCGR3A,ITGA2,ITGAM,ITGB1,ITGB3,ITGB5,LAMP1,PLA2R1,SCARB1,TFRC,THBS1, TLR2,TLR4,VAMP3 |
| hsa04510 | Focal adhesion | 17 | 1.78E-06 | CAV1,CAV3,ITGA1,ITGA2,ITGA2B,ITGA3,ITGA4,ITGA6,ITGB1,ITGB3,ITGB5,PDGFB,PDGFC, PPP1CC,RHOA,THBS1,TLN1 |
| hsa05410 | Hypertrophic cardiomyopathy (HCM) | 11 | 5.39E-06 | ITGA1,ITGA2,ITGA2B,ITGA3,ITGA4,ITGA6,ITGB1,ITGB3,ITGB5,TGFB1,TNF |
| hsa05414 | Dilated cardiomyopathy (DCM) | 11 | 1.06E-05 | ITGA1,ITGA2,ITGA2B,ITGA3,ITGA4,ITGA6,ITGB1,ITGB3,ITGB5,TGFB1,TNF |
| hsa04610 | Complement and coagulation cascades | 10 | 2.59E-05 | C5AR1,CD46,CD59,F10,FGB,FGG,ITGAM,PLAT,PLG,SERPINA5 |
| hsa05152 | Tuberculosis | 14 | 3.45E-05 | CD14,FCGR3A,IFNG,ITGAM,LAMP1,LBP,NOD2,PLA2R1,RHOA,TGFB1,TLR2,TLR4,TNF, TNFRSF1A |
| hsa05144 | Malaria | 8 | 3.81E-05 | IFNG,SDC2,SELP,TGFB1,THBS1,TLR2,TLR4,TNF |
| hsa04015 | Rap1 signaling pathway | 15 | 4.11E-05 | FGF10,GRIN1,GRIN2B,ITGA2B,ITGAM,ITGB1,ITGB3,LPAR1,P2RY1,PDGFB,PDGFC,RALA, RHOA,THBS1,TLN1 |
| hsa04612 | Antigen processing and presentation | 9 | 4.26E-05 | CD4,CD8A,CTSB,HSPA2,HSPA5,IFNG,KLRD1,PDIA3,TNF |
| hsa05140 | Leishmaniasis | 9 | 6.27E-05 | FCGR3A,IFNG,ITGA4,ITGAM,ITGB1,TGFB1,TLR2,TLR4,TNF |
| hsa05412 | Arrhythmogenic right ventricular cardiomyopathy (ARVC) | 9 | 7.35E-05 | ITGA1,ITGA2,ITGA2B,ITGA3,ITGA4,ITGA6,ITGB1,ITGB3,ITGB5 |
| hsa05166 | HTLV-I infection | 16 | 8.91E-05 | FZD1,FZD10,FZD4,FZD9,IL1R1,IL2RA,NRP1,PDGFB,TGFB1,TGFBR2,TLN1,TNF,TNFRSF1A, WNT3A,WNT5A,WNT6 |
| hsa04310 | Wnt signaling pathway | 12 | 9.37E-05 | FZD1,FZD10,FZD4,FZD9,MMP7,PSEN1,RHOA,SFRP1,SFRP4,WNT3A,WNT5A,WNT6 |
| hsa04611 | Platelet activation | 11 | 0.00012 | FGB,FGG,ITGA2,ITGA2B,ITGB1,ITGB3,P2RY1,P2RY12,PPP1CC,RHOA,TLN1 |
| hsa04390 | Hippo signaling pathway | 12 | 0.00015 | BMPR1A,BMPR2,FZD1,FZD10,FZD4,FZD9,PPP1CC,TGFB1,TGFBR2,WNT3A,WNT5A,WNT6 |
| hsa04350 | TGF-beta signaling pathway | 9 | 0.00016 | ACVR2A,BMPR1A,BMPR2,IFNG,RHOA,TGFB1,TGFBR2,THBS1,TNF |
| hsa05217 | Basal cell carcinoma | 8 | 0.00016 | FZD1,FZD10,FZD4,FZD9,HHIP,WNT3A,WNT5A,WNT6 |
| hsa05321 | Inflammatory bowel disease (IBD) | 8 | 0.00016 | IFNG,IL13,IL17A,NOD2,TGFB1,TLR2,TLR4,TNF |
| hsa05162 | Measles | 11 | 0.0002 | CD46,FAS,FASLG,HSPA2,IFNG,IL13,IL2RA,SLAMF1,TLR2,TLR4,TNFRSF10A |
| hsa04360 | Axon guidance | 12 | 0.00042 | BMPR2,EPHB6,ITGB1,NRP1,PLXNB2,RHOA,ROBO2,SEMA7A,SSH1,TRPC4,UNC5D,WNT5A |
| hsa05226 | Gastric cancer | 11 | 0.00042 | ABCB1,FGF10,FZD1,FZD10,FZD4,FZD9,TGFB1,TGFBR2,WNT3A,WNT5A,WNT6 |
| hsa05332 | Graft-versus-host disease | 6 | 0.00042 | CD86,FAS,FASLG,IFNG,KLRD1,TNF |
| hsa04150 | mTOR signaling pathway | 11 | 0.00043 | FZD1,FZD10,FZD4,FZD9,RHOA,SLC3A2,TNF,TNFRSF1A,WNT3A,WNT5A,WNT6 |
| hsa05142 | Chagas disease (American trypanosomiasis) | 9 | 0.00053 | FAS,FASLG,IFNG,TGFB1,TGFBR2,TLR2,TLR4,TNF,TNFRSF1A |
| hsa05323 | Rheumatoid arthritis | 8 | 0.00084 | CD86,CTLA4,IFNG,IL17A,TGFB1,TLR2,TLR4,TNF |
| hsa05145 | Toxoplasmosis | 9 | 0.00086 | HSPA2,IFNG,ITGA6,ITGB1,TGFB1,TLR2,TLR4,TNF,TNFRSF1A |
| hsa04550 | Signaling pathways regulating pluripotency of stem cells | 10 | 0.00099 | ACVR2A,BMPR1A,BMPR2,FZD1,FZD10,FZD4,FZD9,WNT3A,WNT5A,WNT6 |
| hsa04979 | Cholesterol metabolism | 6 | 0.0014 | ABCA1,ANGPTL3,LIPC,LPL,SCARB1,SORT1 |
| hsa05146 | Amoebiasis | 8 | 0.0015 | CD14,IFNG,IL1R1,ITGAM,TGFB1,TLR2,TLR4,TNF |
| hsa04080 | Neuroactive ligand-receptor interaction | 14 | 0.0016 | ADCYAP1R1,C5AR1,CHRNA4,GHRHR,GHSR,GLRA1,GRIN1,GRIN2B,KISS1R,LPAR1,MAS1, NTSR1,P2RY1,PLG |
| hsa04380 | Osteoclast differentiation | 9 | 0.0018 | FCGR3A,IFNG,IL1R1,ITGB3,LILRB2,TGFB1,TGFBR2,TNF,TNFRSF1A |
| hsa04650 | Natural killer cell mediated cytotoxicity | 9 | 0.0018 | FAS,FASLG,FCGR3A,IFNG,KLRD1,MICA,TNF,TNFRSF10A,ULBP2 |
| hsa04660 | T cell receptor signaling pathway | 8 | 0.0018 | CD4,CD8A,CTLA4,IFNG,PDCD1,PTPRC,RHOA,TNF |
| hsa04934 | Cushing's syndrome | 10 | 0.0018 | FZD1,FZD10,FZD4,FZD9,KCNK2,MEN1,SCARB1,WNT3A,WNT5A,WNT6 |
| hsa05150 | Staphylococcus aureus infection | 6 | 0.0018 | C5AR1,FCGR3A,FGG,ITGAM,PLG,SELP |
| hsa04659 | Th17 cell differentiation | 8 | 0.0021 | CD4,IFNG,IL17A,IL1R1,IL2RA,IL6R,TGFB1,TGFBR2 |
| hsa05134 | Legionellosis | 6 | 0.0021 | CD14,HSPA2,ITGAM,TLR2,TLR4,TNF |
| hsa05330 | Allograft rejection | 5 | 0.0021 | CD86,FAS,FASLG,IFNG,TNF |
| hsa04010 | MAPK signaling pathway | 14 | 0.0026 | CD14,FAS,FASLG,FGF10,FLT3LG,HSPA2,IL1R1,PDGFB,PDGFC,TGFA,TGFB1,TGFBR2,TNF, TNFRSF1A |
| hsa05225 | Hepatocellular carcinoma | 10 | 0.0026 | FZD1,FZD10,FZD4,FZD9,TGFA,TGFB1,TGFBR2,WNT3A,WNT5A,WNT6 |
| hsa05340 | Primary immunodeficiency | 5 | 0.0026 | ADA,CD4,CD8A,IL7R,PTPRC |
| hsa04621 | NOD-like receptor signaling pathway | 10 | 0.0028 | ANTXR1,ANTXR2,CTSB,GPRC6A,NOD2,PSTPIP1,RHOA,TLR4,TNF,TRPV2 |
| hsa05010 | Alzheimer's disease | 10 | 0.003 | ADAM17,BACE2,FAS,GRIN1,GRIN2B,IDE,LPL,PSEN1,TNF,TNFRSF1A |
| hsa05132 | Salmonella infection | 7 | 0.003 | CD14,IFNG,LBP,MYH9,PKN1,PKN2,TLR4 |
| hsa05164 | Influenza A | 10 | 0.003 | FAS,FASLG,FURIN,HSPA2,IFNG,PLG,TLR4,TNF,TNFRSF10A,TNFRSF1A |
| hsa04940 | Type I diabetes mellitus | 5 | 0.0032 | CD86,FAS,FASLG,IFNG,TNF |
| hsa04977 | Vitamin digestion and absorption | 4 | 0.004 | CUBN,FOLH1,SCARB1,SLC46A1 |
| hsa04672 | Intestinal immune network for IgA production | 5 | 0.0045 | CCR10,CCR9,CD86,ITGA4,TGFB1 |
| hsa04062 | Chemokine signaling pathway | 10 | 0.0046 | CCR1,CCR10,CCR2,CCR7,CCR9,CX3CL1,CXCL9,CXCR3,CXCR5,RHOA |
| hsa04916 | Melanogenesis | 7 | 0.0062 | FZD1,FZD10,FZD4,FZD9,WNT3A,WNT5A,WNT6 |
| hsa05100 | Bacterial invasion of epithelial cells | 6 | 0.0063 | CAV1,CAV3,ITGB1,RHOA,SEPT12,SEPT2 |
| hsa05320 | Autoimmune thyroid disease | 5 | 0.0065 | CD86,CTLA4,FAS,FASLG,TPO |
| hsa05133 | Pertussis | 6 | 0.0069 | CD14,ITGAM,ITGB1,RHOA,TLR4,TNF |
| hsa04620 | Toll-like receptor signaling pathway | 7 | 0.0072 | CD14,CD86,CXCL9,LBP,TLR2,TLR4,TNF |

**Supplementary References**

1. Elliott DA, Braam SR, Koutsis K, Ng ES, Jenny R, Lagerqvist EL, Biben C, Hatzistavrou T, Hirst CE, Yu QC, Skelton RJ, Ward-van Oostwaard D, Lim SM, Khammy O, Li X, Hawes SM, Davis RP, Goulburn AL, Passier R, Prall OW, Haynes JM, Pouton CW, Kaye DM, Mummery CL, Elefanty AG and Stanley EG. NKX2-5(eGFP/w) hESCs for isolation of human cardiac progenitors and cardiomyocytes. *Nature methods*. 2011;8:1037-40.

2. Rim YA, Park N, Nam Y, Ham DS, Kim JW, Ha HY, Jung JW, Jung SM, Baek IC, Kim SY, Kim TG, Song J, Lee J, Park SH, Chung NG, Yoon KH and Ju JH. [Korean-homozygous HLA-iPSC]-Recent progress of national banking project on homozygous HLA-typed induced pluripotent stem cells in South Korea. *Journal of tissue engineering and regenerative medicine*. 2018;12:e1531-e1536.

3. Burridge PW, Matsa E, Shukla P, Lin ZC, Churko JM, Ebert AD, Lan F, Diecke S, Huber B, Mordwinkin NM, Plews JR, Abilez OJ, Cui B, Gold JD and Wu JC. Chemically defined generation of human cardiomyocytes. *Nature methods*. 2014;11:855-60.

4. Jo HY, Han HW, Jung I, Ju JH, Park SJ, Moon S, Geum D, Kim H, Park HJ, Kim S, Stacey GN, Koo SK, Park MH and Kim JH. Development of genetic quality tests for good manufacturing practice-compliant induced pluripotent stem cells and their derivatives. *Scientific reports*. 2020;10:3939.

5. Burridge PW, Holmstrom A and Wu JC. Chemically Defined Culture and Cardiomyocyte Differentiation of Human Pluripotent Stem Cells. *Current protocols in human genetics*. 2015;87:21 3 1-21 3 15.

6. Trapnell C, Pachter L and Salzberg SL. TopHat: discovering splice junctions with RNA-Seq. *Bioinformatics*. 2009;25:1105-11.

7. Langmead B, Trapnell C, Pop M and Salzberg SL. Ultrafast and memory-efficient alignment of short DNA sequences to the human genome. *Genome biology*. 2009;10:R25.

8. Trapnell C, Williams BA, Pertea G, Mortazavi A, Kwan G, van Baren MJ, Salzberg SL, Wold BJ and Pachter L. Transcript assembly and quantification by RNA-Seq reveals unannotated transcripts and isoform switching during cell differentiation. *Nature biotechnology*. 2010;28:511-5.

9. Choi SW, Lee HA, Moon SH, Park SJ, Kim HJ, Kim KS, Zhang YH, Youm JB and Kim SJ. Spontaneous inward currents reflecting oscillatory activation of Na(+)/Ca(2)(+) exchangers in human embryonic stem cell-derived cardiomyocytes. *Pflugers Archiv : European journal of physiology*. 2016;468:609-22.

10. Wimmer RA, Leopoldi A, Aichinger M, Kerjaschki D and Penninger JM. Generation of blood vessel organoids from human pluripotent stem cells. *Nature protocols*. 2019;14:3082-3100.

11. Moon SH, Kang SW, Park SJ, Bae D, Kim SJ, Lee HA, Kim KS, Hong KS, Kim JS, Do JT, Byun KH and Chung HM. The use of aggregates of purified cardiomyocytes derived from human ESCs for functional engraftment after myocardial infarction. *Biomaterials*. 2013;34:4013-4026.

12. Park SJ, Kim RY, Park BW, Lee S, Choi SW, Park JH, Choi JJ, Kim SW, Jang J, Cho DW, Chung HM, Moon SH, Ban K and Park HJ. Dual stem cell therapy synergistically improves cardiac function and vascular regeneration following myocardial infarction. *Nature communications*. 2019;10:3123.

13. Tang XL, Li Q, Rokosh G, Sanganalmath SK, Chen N, Ou Q, Stowers H, Hunt G and Bolli R. Long-Term Outcome of Administration of c-kit(POS) Cardiac Progenitor Cells After Acute Myocardial Infarction: Transplanted Cells Do not Become Cardiomyocytes, but Structural and Functional Improvement and Proliferation of Endogenous Cells Persist for at Least One Year. *Circulation research*. 2016;118:1091-105.

14. Xu W, Barrientos T, Mao L, Rockman HA, Sauve AA and Andrews NC. Lethal Cardiomyopathy in Mice Lacking Transferrin Receptor in the Heart. *Cell reports*. 2015;13:533-545.

15. Nguyen PK, Neofytou E, Rhee JW and Wu JC. Potential Strategies to Address the Major Clinical Barriers Facing Stem Cell Regenerative Therapy for Cardiovascular Disease: A Review. *JAMA cardiology*. 2016;1:953-962.

16. Thavapalachandran S, Grieve SM, Hume RD, Le TYL, Raguram K, Hudson JE, Pouliopoulos J, Figtree GA, Dye RP, Barry AM, Brown P, Lu J, Coffey S, Kesteven SH, Mills RJ, Rashid FN, Taran E, Kovoor P, Thomas L, Denniss AR, Kizana E, Asli NS, Xaymardan M, Feneley MP, Graham RM, Harvey RP and Chong JJH. Platelet-derived growth factor-AB improves scar mechanics and vascularity after myocardial infarction. *Science translational medicine*. 2020;12.

17. Liu YW, Chen B, Yang X, Fugate JA, Kalucki FA, Futakuchi-Tsuchida A, Couture L, Vogel KW, Astley CA, Baldessari A, Ogle J, Don CW, Steinberg ZL, Seslar SP, Tuck SA, Tsuchida H, Naumova AV, Dupras SK, Lyu MS, Lee J, Hailey DW, Reinecke H, Pabon L, Fryer BH, MacLellan WR, Thies RS and Murry CE. Human embryonic stem cell-derived cardiomyocytes restore function in infarcted hearts of non-human primates. *Nature biotechnology*. 2018;36:597-605.

18. Keeley TP and Mann GE. Defining Physiological Normoxia for Improved Translation of Cell Physiology to Animal Models and Humans. *Physiological reviews*. 2019;99:161-234.

19. Langan LM, Dodd NJ, Owen SF, Purcell WM, Jackson SK and Jha AN. Direct Measurements of Oxygen Gradients in Spheroid Culture System Using Electron Parametric Resonance Oximetry. *PloS one*. 2016;11:e0149492.

20. Moon SH, Ju J, Park SJ, Bae D, Chung HM and Lee SH. Optimizing human embryonic stem cells differentiation efficiency by screening size-tunable homogenous embryoid bodies. *Biomaterials*. 2014;35:5987-97.

21. Adine C, Ng KK, Rungarunlert S, Souza GR and Ferreira JN. Engineering innervated secretory epithelial organoids by magnetic three-dimensional bioprinting for stimulating epithelial growth in salivary glands. *Biomaterials*. 2018;180:52-66.

22. Bolli R, Hare JM, March KL, Pepine CJ, Willerson JT, Perin EC, Yang PC, Henry TD, Traverse JH, Mitrani RD, Khan A, Hernandez-Schulman I, Taylor DA, DiFede DL, Lima JAC, Chugh A, Loughran J, Vojvodic RW, Sayre SL, Bettencourt J, Cohen M, Moye L, Ebert RF, Simari RD and Cardiovascular Cell Therapy Research N. Rationale and Design of the CONCERT-HF Trial (Combination of Mesenchymal and c-kit(+) Cardiac Stem Cells As Regenerative Therapy for Heart Failure). *Circulation research*. 2018;122:1703-1715.

23. Cambria E, Pasqualini FS, Wolint P, Gunter J, Steiger J, Bopp A, Hoerstrup SP and Emmert MY. Translational cardiac stem cell therapy: advancing from first-generation to next-generation cell types. *NPJ Regenerative medicine*. 2017;2:17.

24. Menasche P, Vanneaux V, Hagege A, Bel A, Cholley B, Parouchev A, Cacciapuoti I, Al-Daccak R, Benhamouda N, Blons H, Agbulut O, Tosca L, Trouvin JH, Fabreguettes JR, Bellamy V, Charron D, Tartour E, Tachdjian G, Desnos M and Larghero J. Transplantation of Human Embryonic Stem Cell-Derived Cardiovascular Progenitors for Severe Ischemic Left Ventricular Dysfunction. *Journal of the American College of Cardiology*. 2018;71:429-438.

25. Yui Y. Concerns on a new therapy for severe heart failure using cell sheets with skeletal muscle or myocardial cells from iPS cells in Japan. *NPJ Regenerative medicine*. 2018;3:7.

26. Eguchi A, Naito Y, Iwasaku T, Okuhara Y, Morisawa D, Sawada H, Nishimura K, Oboshi M, Fujii K, Mano T, Masuyama T and Hirotani S. Association of dietary iron restriction with left ventricular remodeling after myocardial infarction in mice. *Heart and vessels*. 2016;31:222-9.

27. Cai W, Zhang J, de Lange WJ, Gregorich ZR, Karp H, Farrell ET, Mitchell SD, Tucholski T, Lin Z, Biermann M, McIlwain SJ, Ralphe JC, Kamp TJ and Ge Y. An Unbiased Proteomics Method to Assess the Maturation of Human Pluripotent Stem Cell-Derived Cardiomyocytes. *Circulation research*. 2019;125:936-953.

28. Chong JJ, Yang X, Don CW, Minami E, Liu YW, Weyers JJ, Mahoney WM, Van Biber B, Cook SM, Palpant NJ, Gantz JA, Fugate JA, Muskheli V, Gough GM, Vogel KW, Astley CA, Hotchkiss CE, Baldessari A, Pabon L, Reinecke H, Gill EA, Nelson V, Kiem HP, Laflamme MA and Murry CE. Human embryonic-stem-cell-derived cardiomyocytes regenerate

29. Laflamme MA, Chen KY, Naumova AV, Muskheli V, Fugate JA, Dupras SK, Reinecke H, Xu C, Hassanipour M, Police S, O'Sullivan C, Collins L, Chen Y, Minami E, Gill EA, Ueno S, Yuan C, Gold J and Murry CE. Cardiomyocytes derived from human embryonic stem cells in pro-survival factors enhance function of infarcted rat hearts. *Nature biotechnology*. 2007;25:1015-24.

non-human primate hearts. *Nature*. 2014;510:273-7.

30. Romagnuolo R, Masoudpour H, Porta-Sanchez A, Qiang B, Barry J, Laskary A, Qi X, Masse S, Magtibay K, Kawajiri H, Wu J, Valdman Sadikov T, Rothberg J, Panchalingam KM, Titus E, Li RK, Zandstra PW, Wright GA, Nanthakumar K, Ghugre NR, Keller G and Laflamme MA. Human Embryonic Stem Cell-Derived Cardiomyocytes Regenerate the Infarcted Pig Heart but Induce Ventricular Tachyarrhythmias. *Stem cell reports*. 2019;12:967-981.

31. Jang J, Park HJ, Kim SW, Kim H, Park JY, Na SJ, Kim HJ, Park MN, Choi SH, Park SH, Kim SW, Kwon SM, Kim PJ and Cho DW. 3D printed complex tissue construct using stem cell-laden decellularized extracellular matrix bioinks for cardiac repair. *Biomaterials*. 2017;112:264-274.
